# Chemoresistome mapping in individual breast cancer patients unravels diversity in dynamic transcriptional adaptation

**DOI:** 10.1002/1878-0261.70030

**Published:** 2025-04-28

**Authors:** Maya Dadiani, Gilgi Friedlander, Gili Perry, Nora Balint‐Lahat, Shlomit Gilad, Dana Morzaev‐Sulzbach, Anjana Shenoy, Noa Bossel Ben‐Moshe, Anya Pavlovsky, Rinat Bernstein‐Molho, Eytan Domany, Iris Barshack, Tamar Geiger, Bella Kaufman, Einav Nili Gal‐Yam

**Affiliations:** ^1^ Cancer Research Center Sheba Medical Center Ramat Gan Israel; ^2^ The Nehemia Rubin Excellence in Biomedical Research The TELEM Program Ramat Gen Israel; ^3^ Mantoux Bioinformatics Institute, The Nancy & Stephen Grand Israel National Center for Personalized Medicine Weizmann Institute of Science Rehovot Israel; ^4^ Pathology Institute Sheba Medical Center Ramat Gan Israel; ^5^ The Nancy & Stephen Grand Israel National Center for Personalized Medicine Weizmann Institute of Science Rehovot Israel; ^6^ Sackler Faculty of Medicine Tel Aviv University Tel‐Aviv Israel; ^7^ Department of Molecular Cell Biology Weizmann Institute of Science Rehovot Israel; ^8^ Department of Physics of Complex Systems Weizmann Institute of Science Rehovot Israel; ^9^ The Suzanne Levy‐Gertner Oncogenetics Unit Sheba Medical Center Ramat Gan Israel; ^10^ Oncology Institute Sheba Medical Center Ramat Gan Israel; ^11^ The Dr. Pinchas Borenstein Talpiot Medical Leadership Program Sheba Medical Center Ramat Gan Israel

**Keywords:** breast cancer, chemotherapy, resistance, transcriptional adaptation

## Abstract

Nongenetic adaptive resistance to chemotherapy, driven by transcriptional rewiring, is emerging as a significant mechanism in tumor survival. In this study we combined longitudinal transcriptomics with temporal pattern analysis to investigate patient‐specific mechanisms underlying acquired resistance in breast cancer. Matched tumor biopsies (pretreatment, posttreatment, and adjacent normal) were collected from breast cancer patients who received neoadjuvant chemotherapy. Transcriptomes were analyzed by longitudinal gene‐pattern classification to track patient‐specific gene expression alterations that occur during treatment. Our findings reveal that resistance‐associated genes were already dysregulated in primary tumors, suggesting the presence of a preexisting drug‐tolerant state. While each patient displayed unique resistance‐associated gene rewiring, these alterations converged into a limited number of dysregulated functional modules. Notably, patients receiving the same treatment exhibited distinct rewiring of genes and pathways, revealing parallel, individualized routes to resistance. In conclusion, we propose that tumor cells survive chemotherapy by sustaining or amplifying a preexisting drug‐tolerant state that circumvents drug action. We suggest that individualized “chemoresistome maps” could identify cancer vulnerabilities and inform personalized therapeutic strategies to overcome or prevent resistance.

## Graphical abstract

This study used longitudinal transcriptomics and gene‐pattern classification to uncover patient‐specific mechanisms of chemotherapy resistance in breast cancer. Findings reveal preexisting drug‐tolerant states in primary tumors and diverse gene rewiring patterns across patients, converging on a few dysregulated functional modules. Despite receiving the same treatments, patients develop unique, parallel pathways to resistance, highlighting individualized therapeutic complexity.

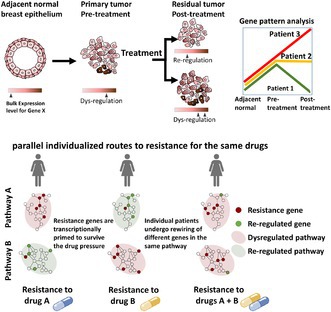

**Abbreviations**
ADC: antibody‐drug‐conjugates
ECM: extracellular matrix
FFPE: formalin‐fixed paraffin‐embedded
HR+: hormone receptor positive
IRB: institutional review board
MP: Miller–Payne
NAT: neoadjuvant treatment
NBR: normal breast tissue
RCB: residual cancer burden
TNBC: triple negative breast cancer
VST: variance stabilizing transformation

## 1 Introduction

Despite the considerable importance of tumor drug resistance to cancer morbidity and mortality, our comprehension of the various molecular mechanisms involved in resistance is limited [1]. The actual response of an individual patient remains a ‘black box.’ The oncologist cannot accurately predict which tumor, eligible for a chemotherapy, will be eliminated by the drugs, and the intrinsic resistance to chemotherapy remains a substantial enigma. Recently, the prevailing genetic clonal selection, as the main mechanism of resistance, is challenged by emerging indications of nongenetic adaptive mechanisms of drug resistance [2, 3]. Cumulative evidence shows that in response to treatment, tumors adopt a drug‐tolerant state through transcriptional reprogramming [4, 5, 6]. To survive chemotherapy, cancer cells rewire molecular pathways, thereby escaping antiproliferative drugs. However, as cancer is a continuous process, the individual route to resistance in each cancer type and each patient is elusive.

Breast cancer is currently the most common type of female cancer and the second cause of cancer mortality in women [7]. Major international efforts have profiled primary tumors and defined the molecular architecture of breast cancer [8, 9, 10, 11, 12]. It is now clear that breast cancer is a heterogeneous disease, classified into distinct subtypes: hormone receptor positive (HR+), HER2‐positive and triple‐negative breast cancer (TNBC). While novel targeted therapies and immunotherapies are being developed, chemotherapy remains a mainstay of treatment for early high‐risk breast cancer patients, and the backbone of the newly developed antibody‐drug‐conjugates (ADCs) [13]. Thus, uncovering the mechanisms of chemoresistance is a crucial necessity.

To elucidate resistance mechanisms, the ideal way is to perform longitudinal studies, looking at matched pre‐ and posttreated tumors. The neoadjuvant (NAT) preoperative setting provides a unique opportunity to study the effects of chemotherapy on real‐life tumors by the inherent existence of both pretreatment diagnostic biopsy and posttreatment surgery specimen [14]. NATs are widely used for the treatment of high‐risk breast cancer patients and provide predictive and long‐term prognostic information by evaluating tumor chemosensitivity [15]. The quest for biomarkers predicting response or for molecular targets avoiding resistance instigated many studies that inspected post‐NAT residual tumors. While initial studies profiled one timepoint primary tumor sample [16] or residual post‐NAT tumors [17], later studies compared pre‐ and posttreatment tumors [18, 19, 20, 21, 22], resulting in the discovery of treatment‐altered genes and pathways. Yet, most studies were based on statistical differential expression and could not provide a personalized view of resistance. More recent studies inspected time‐course changes following various neoadjuvant treatment modalities, using matched serial sampling of three timepoints: pre‐NAT, short‐term and long‐term posttreatment [6, 14, 23, 24, 25, 26]; nevertheless, the mechanisms underlying nongenetic resistance and the extent of phenotypic diversity between patients following treatment remain elusive. Transcriptional reprogramming in response to chemotherapy can be mediated by a stochastic mechanism that drives nongenetic heterogeneity [27]. It is unclear, yet, whether the nongenetic resistance involves active reprograming of specific genes during treatment or, alternatively, intensify transcriptionally primed genes. To investigate this in clinically relevant samples, we collected a cohort of neoadjuvant breast cancer patients and profiled their transcriptome before and after treatment, compared to the adjacent normal epithelium of the same patient. Thereby, we inspected drug‐induced modulations relative to the deregulation state of the primary tumor vs its matched normal breast. To understand the personal route to resistance, we applied a patient‐oriented pattern analysis algorithm for dealing with longitudinal datasets at the gene level. We previously developed this pattern analysis approach to investigate miRNA expression modulations following recurrence [28], and further applied it to a longitudinal proteomic NAT dataset of breast cancer patients, revealing the involvement of proline biosynthesis in resistance [29].

The analysis of our longitudinal dataset depicted important principles in the adaptive nongenetic mechanism of resistance. By deconstructing the dynamic pattern modulation for each gene per patient, we detected rewiring of simultaneous multiple pathways, albeit in a unique fashion to each patient. Personal chemoresistome mapping can provide insightful mechanistic understanding of therapy resistance with future clinical implication for rational design of treatment plans.

## 2 Methods

### 2.1 Cohort assembly

The cohort consisted of 29 breast cancer patients who underwent neoadjuvant therapy, and did not achieve complete response, resulting in partial response and available residual tumor. Only patients with partial response were included, as we aimed to focus on dynamic changes between the primary tumor and the residual tumor. Clinicopathological information for the cohort is summarized in Table 1 and detailed in Table S1. For each patient we collected triplets of pretreatment tumor (diagnostic biopsy), posttreatment residual tumor (sampled at surgery, ~6–8 months after the pretreatment biopsy), and adjacent normal epithelium (sampled from the posttreatment surgery specimen). Samples were collected as formalin‐fixed paraffin‐embedded (FFPE) tissues from the Sheba Medical Center pathology archive. Importantly, tissue collection and the fixation time are standardized in the pathology department. Moreover, we previously showed that transcriptome profiling from FFPE tissues using our optimized protocol was similar to the transcriptome of the same snap‐frozen tissue [30]. Additional normal breast epithelium samples were collected from breast reduction specimens of six healthy individuals. Overall, a total of 97 samples were included in this study. Two independent pathologists inspected the slides to define tumor cellularity, marking regions for macrodissection to enrich for tumor cellularity in the RNA extraction. Pathologists scored tumor response by Miller–Payne (MP) score [31], which assesses the reduction in tumor cellularity by comparing pretreatment biopsy samples to posttreatment surgical specimens, and by RCB (Residual Cancer Burden class) [32]. Recurrence‐free survival according to Miller–Payne scores was performed by Kaplan–Meier analysis, calculating the log‐rank *P*‐value. The study was approved by the Sheba Medical Center Institutional Review Board (IRB) (approval #8736‐11), in accordance with the principles of the Declaration of Helsinki, and with the Public Health Regulations. The study protocol authorized a full exemption for consent form for anonymized samples. Accordingly, all samples were anonymized as defined in the study protocol.

**Table 1:** Clinicopathological parameters for the entire cohort

Neoadjuvant breast cancer cohort	*n* = 29
Age, median (range)	54 (31–80)
Pathological subtype (*n*, %)	
Triple‐negative (TNBC)	3 (10%)
HER2−/hormone‐receptor positive (HR+)	16 (55%)
HER2+/hormone‐receptor positive (TP)	10 (35%)
T stage at diagnosis (*n*, %)	
T1	3 (10%)
T2	18 (62%)
T3	6 (21%)
T4	2 (7%)
Histopathological classification	
IDC	22 (76%)
IDC + DCIS	4 (14%)
ILC	3 (10%)
LN+ at diagnosis (*n*, %)	
Negative	5 (17%)
Positive	24 (83%)
Neoadjuvant chemotherapy (*n*, %)	
AC‐T	14 (48%)
AC‐TH	11^a^ (38%)
Partial (AC or A‐T or C‐T)	4 (14%)
Response to therapy, RCB class (*n*, %)	
RCB‐I	1 (3%)
RCB‐II	13 (45%)
RCB‐III	15 (52%)
Response to therapy, Miller–Payne grade (*n*, %)	
MP‐1	5 (17%)
MP‐2	10 (35%)
MP‐3	13 (45%)
MP‐4	1 (3%)
Recurrence (*n*, %)	
Loco‐regional	3 (10%)
Metastatic	4 (14%)
Median RFS, years (range)	6 (1–11)
Median follow‐up from diagnosis, years (range)	7 (1–11)

LN, lymph nodes; MP, Miller–Payne; RCB, residual cancer burden; RFS, recurrence free survival. Chemotherapies: A – adriamycin (doxorubicin), C – cyclophosphamide, T – taxol (paclitaxel), H – herceptin (most HER2+ patients were treated before Pertuzumab was included in the standard of care treatment).

^a^ One patient had bilateral tumors (HR+ was sampled and HER2+ was not sampled), hence received herceptin.

### 2.2 mRNAseq library preparation

FFPE tumor samples were macrodissected to enrich for tumor cellularity. The 5‐μm sections were deparaffinized at 90 °C for 5 min. Total RNA as well as DNA were extracted using nucleic acid isolation kit (AllPrep, Qiagen, Hilden, Germany) according to the protocol instructions. RNA concentrations were determined by Qubit fluorometer (Thermofisher Scientific, Waltham, MA, USA). Whole transcriptome profiling of the archived samples was accomplished by a reliable and cost‐effective mRNAseq procedure that we previously optimized for FFPE samples [30]. Briefly, mRNA‐seq libraries were constructed by Truseq RNA sample preparation kit v2 (Illumina, San Diego, CA, USA). Library size was evaluated by Tapestation (Agilent, Santa Clara, CA, USA). Primer dimers were eliminated using 1× Agencourt RNAClean XP Beads. Sequencing libraries were constructed with the TruSeq SBS Kit and multiplexed to run 6–10 samples per lane with a read length of 60 bp single‐end run in an Illumina HiSeq V4 instrument. The median sequencing depth was ~27 million reads per sample.

### 2.3 Sequence alignment and mapping

Illumina adapters and PolyA/T stretches were trimmed using cutadapt [33]. Reads shorter than 30 bp were discarded. The reads were mapped to the human genome (GRCh38) using star v2.4.2a [34]. Overall, mapping of reads to the genome was high, with an average of 87% reads being mapped and 61% of the uniquely mapped reads being counted. Median uniquely mapped reads were 10.7 million reads. Counting proceeded over genes annotated in Ensembl release 88, using htseq‐count [35]. Out of the 97 samples, four normal samples failed the library preparation steps due to low RNA quantity. An additional six samples failed ‐quality control for mapping and three samples were excluded due to low tumor cellularity. Overall, 84 samples from 29 patients were included in the general analysis. Finally, 23 patients had complete triplet samples that passed all data QC and were used for pattern analysis (Fig. S1). A pipeline was constructed using snakemake [36]. Data analysis was mostly done by R language [37] using the specified packages.

### 2.4 mRNAseq data evaluation

The dynamic range of the mRNAseq data was correlated with pathological scoring values of immunohistochemistry staining for: ER, PR, HER2, and KI67 when available (Fig. S2A,B). The percentage of Ki67 positive cells were assessed blinded to the expression data using the automated Virtuoso image analysis algorithm (Ventana Medical Systems, Tucson, AZ, USA). The dynamic range of the mRNAseq data at the highest pathological scores for the receptors was much higher than the immunohistochemistry staining levels (Fig. S2C). To evaluate the purity of the tumor samples from the mRNAseq counts, we used the ESTIMATE algorithm [38] and compared it to tumor purity, estimated by two independent pathologists after the macrodissection. There was a good agreement between the pathological evaluation and the purity calculated by the ESTIMATE algorithm.

### 2.5 Differential expression analysis

To analyze the differential expression between matched sample types (normal—pretreatment—posttreatment) we utilized the deseq2 package [39] with the betaPrior, cooksCutoff, and independent Filtering parameters set to False. The patient information was added to the deseq2 model to control its effect. Raw *P* values were adjusted for multiple testing using the procedure of Benjamini and Hochberg. To estimate the number of differentially expressed genes between each pair of sample types, the following contrasts were defined: posttreatment tumor vs normal, pretreatment tumor vs normal, and posttreatment vs pretreatment. The genes were filtered to keep genes with an absolute fold change above 2, adjusted *P*‐value below or equal to 0.05, and a count of at least 100 in at least one of the samples. About 6900 genes passed this threshold. Molecular subtype was calculated from the mRNAseq data by the genefu package [40] using the SCMOD2 subtyping algorithms [41] (detailed in Table S1).

### 2.6 Pattern classification

To analyze the modification of genes across the three timepoints (normal, pretreatment, and posttreatment), we defined all possible eight theoretical patterns (Fig. 2C) as previously published [28]. To assign a pattern for each gene we correlated the changes in expression values, calculated by deseq2 Variance Stabilizing Transformation (VST), to the theoretical patterns. VST calculates sample geometric means, estimates dispersions for each gene, fits a mean‐dispersion trend, and then transforms the data to stabilize the variance across the mean. Each gene was assigned to a theoretical pattern (according to the maximal correlation). Genes were considered as classified into one main pattern if they were classified to a single pattern in at least 50% of patients. Genes were considered as classified into bimodal patterns if the gene was classified to each of the two patterns in at least 30% of the patients and each of the two patterns was observed at least in five patients, based on the distribution of the pattern frequencies.

### 2.7 Defining genes associated with response

For genes with main persisted patterns: classification of P3 or P3 + P7 or P4 or P4 + P8 in more than 50% of patients. For genes with bimodal patterns, we compared the Miller–Payne score of the patients with “good response” patterns (P1 or P2) versus scores of patients with “poor response” patterns (P3, P7 or P4, P8). The Wilcoxon ranks sum test was used to calculate the significance of the pattern association with response. The test was calculated for each gene cluster separately: P1 vs P3/P7 and P2 vs P4/P8 (Fig. 3B). The test was repeated for HR+ patients only.

### 2.8 Pathifier algorithm

**Fig. 1:**
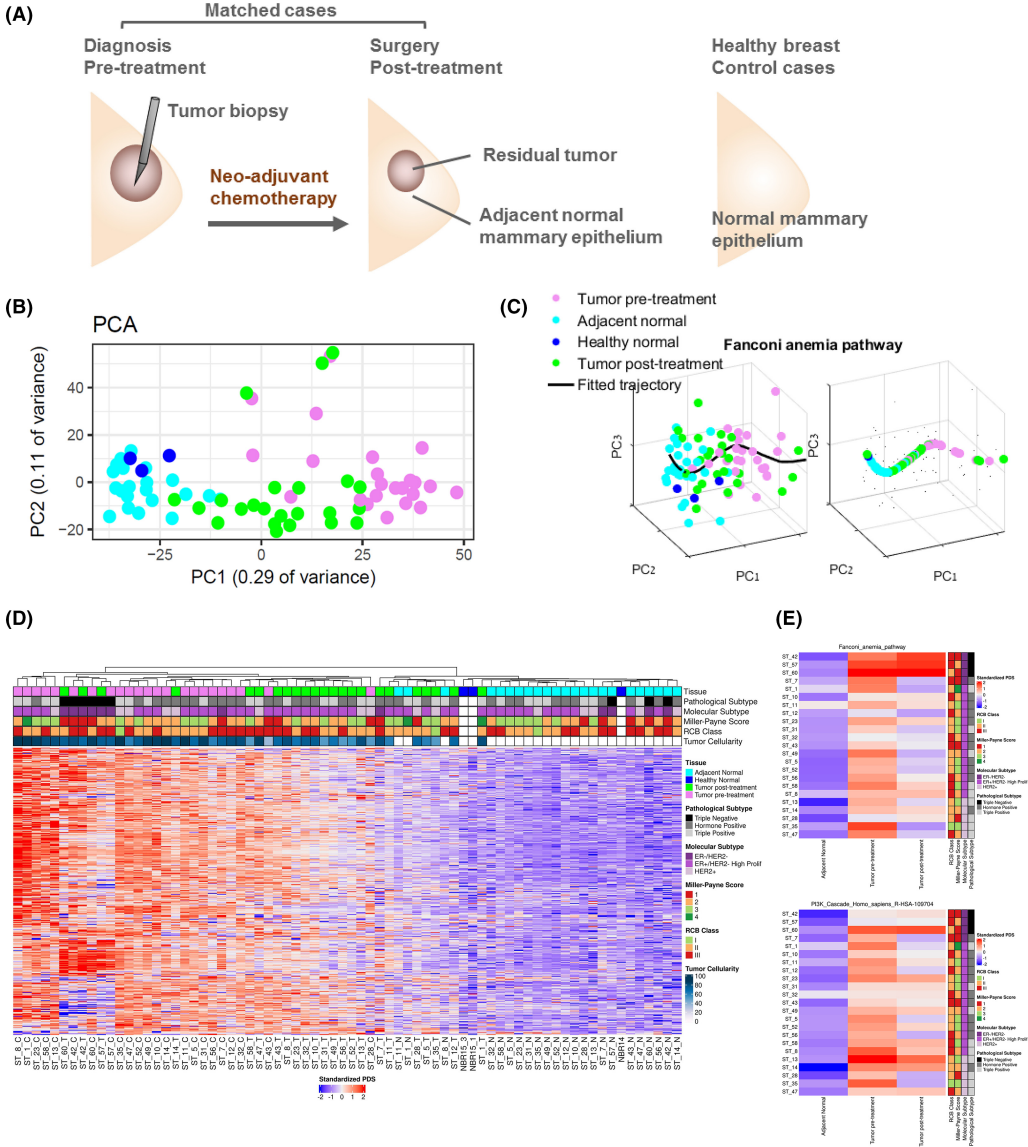
Longitudinal transcriptomics of matched samples from breast cancer patients receiving neoadjuvant chemotherapy. (A) Archived samples from 29 breast cancer patients undergoing neoadjuvant therapy were collected. From each patient we analyzed matched pretreatment, posttreatment, and adjacent normal breast epithelia. In addition, normal breast samples from three healthy women were included. (B) Principal components analysis of the 1000 most variable genes from the whole transcriptome mRNAseq data. Gene expression clustered according to sample type. Adjacent normal breast samples were similar to normal mammary from healthy women. (C) An example of calculating pathway deregulation score (PDS) using the Pathifier algorithm for a selected pathway from the KEGG dataset. The principal curve (black) is going through the cloud of points representing the various samples. The samples (colored according to their type) are projected onto the curve. (D) Pathway Deregulation Scores (PDS) were calculated for each sample per pathway using the Pathifier algorithm. Each row represents a different pathway (KEGG, Reactome, and GO), for which a PDS relative to all normal samples (healthy + adjacent) is calculated. Each column represents a different sample in individual patients. Notably, healthy normal breast tissue (NBR) is very similar to the adjacent normal tissues (N), as shown by their overall low PDS (blue). Tumor samples pretreatment show the most deregulated scores, as shown by their overall red levels. Tumor samples posttreatment are variable. (E) Temporal modulations in deregulation scores per‐patient for two representative pathways. RCB, residual cancer burden.

We used the Pathifier algorithm [42, 43] to calculate pathways deregulation scores (PDS) for each patient's sample for a combined list of pathways databases: GO_biological process (2018), KEGG (2019), and Reactome (2016), all downloaded from the EnricheR website [44]. Briefly, Pathifier analyzes each pathway, and assigns to each sample i and pathway P a score that estimates the extent to which the behavior of pathway P deviates from normal samples. To determine PDS, we used the normalized counts of all 97 samples. For each pathway, PDS is defined as the distance of each sample from its projection on the calculated principal curve of all samples to the projection of the normal samples (Fig. 1C). Normal samples were defined as both the healthy normal and the adjacent normal samples.

### 2.9 Pathway enrichment analysis and resistance score calculations

Pathway enrichment analysis for genes that were significantly associated with response (Wilcoxon rank‐sum test, *P*‐value < 0.05) was calculated using clusterprofiler package [45], using pathways from the following databases: GO_biological process (2018), KEGG (2019), and Reactome (2016) (downloaded from the EnrichR database [44] website). For each enriched pathway, we calculated resistance score per patient and a *P*‐value. The resistance score is defined as the fraction of “resistance genes” (genes with patterns P3, P4, P7, or P8) in the pathway. Only genes that were significantly differentially expressed were considered. The *P*‐value for this score was calculated by bootstrapping random sets of genes of the same size of the pathway (sampled 10 000 times). For each patient in each pathway, the *P*‐value was calculated by dividing the number of random scores, which were greater or equal to the observed score, by the number of permutations. To reduce subtype bias, pathway enrichment was performed twice: for the entire cohort of patients (23 patients) and for HR+ patients (20 patients), excluding three TNBCs. We then intersected the enriched pathways between the two groups to reach a robust list of pathways (33 pathways). To compensate for patient diversity, we added to the shared list the top five most significant pathways unique to each group, resulting in 43 pathways. The enriched pathways were then curated to a final list of 24 pathways, accounting for significance and functional redundancy. These pathways were divided into functional modules. For a simple representation of resistance modules in each patient, we generated personal chemoresistome maps. Circular barplots depicting the resistance score and *P*‐value in each module were plotted for each patient.

### 2.10 STRING analysis

To identify the functional network of the resistance genes (254 genes, *P*‐value by Wilcoxon rank test < 0.05), we ran STRING analysis (v. 11.5) [46]. The interaction score was set to the highest confidence (0.9). *K*‐means clustering (defined for 11 clusters) was used to identify functional categories. Functional categories were defined by enrichment of each cluster by reactome and the GO biological process. For visualization of resistance genes in each pathway, all genes in the pathway were uploaded to the STRING's payload mechanism and a color‐code was specified for a halo around the STRING nodes.

### 2.11 Genetic test for pathogenic variants

Genotyping for recurrent pathogenic variants (PVs) in BRCA1/2 and additional cancer susceptibility genes was performed for 17 patients lacking prior genetic information. The gene panel tested 51 variants, including 30 BRCA1/2 PVs and 21 additional variants known in Jewish and non‐Jewish populations. Testing was conducted using the NanoCHIP XL system (Savyon Diagnostics, Ashdod, Israel). A comprehensive list of the tested variants is provided in reference [47].

## 3 Results

### 3.1 Longitudinal dataset of breast cancer patients receiving neoadjuvant chemotherapy

Matched archived samples (pretreatment tumor, posttreatment tumor, and adjacent normal breast epithelia) were collected from a cohort of breast cancer patients who underwent neoadjuvant chemotherapy (Fig. 1A). Importantly, samples were enriched for tumor cells by macrodissection, and most samples included at least 70% tumor cells, as scored by pathologists. The full description of clinicopathological characteristics is summarized in Table 1 and detailed in Table S1, including tumor cellularity. Chemotherapy treatment included a standard‐of‐care sequential doxorubicin and cyclophosphamide followed by paclitaxel, and additional Herceptin for HER2+ patients. Posttreatment samples were scored for response by both the Miller–Payne (MP) score [31] and RCB grade [32]. A whole‐transcriptome mRNAseq dataset was generated, using our optimized mRNAseq methodology, for archived FFPE samples [30]. A flow diagram presents the cases included in the analysis (Fig. S1).

### 3.2 Different sample types cluster together showing distinct regulation states

Expression‐based principal component analysis (PCA) of all samples demonstrated clustering of the samples according to sample type (pre‐ or posttreatment tumors and normal epithelium) (Fig. 1B). Posttreatment samples were positioned between the adjacent normal tissues and the cancerous pretreatment tissues, possibly reflecting the range of response to treatment. Notably, the healthy normal breasts were clustered with the adjacent normal samples of cancer patients (Fig. 1B), shown also as a heatmap of the 1000 most differentially expressed genes between tumor and normal (Fig. S3). This observation supports our assumption that the adjacent normal breast tissues of cancer patients reliably represent expression of normal breast tissue.

The overall modulations occurring through the course of disease and therapy were estimated by calculating the pathway deregulation score (PDS) for the various samples, relative to all normal samples using Pathifier [42, 43]. This algorithm calculates a PDS for each sample, based on the distance from the projection of the cloud of points on a principal curve (representative pathway exemplified in Fig. 1C). PDS was calculated for a combined list of pathways (GO_biological process, KEGG, and Reactome). A bird's‐eye view of the deregulation scores is shown as a heatmap of all pathways across all samples, ordered by hierarchical clustering (Fig. 1D). Notably, adjacent normal tissues were clustered with healthy normal breast tissues, indicating that these posttreatment normal samples largely represent normal breast epithelium. Pretreatment tumors exhibited the most deregulated scores, whereas posttreatment tumors were variable, some were clustered with pretreatment samples, and others with adjacent normal, irrespective of tumor cellularity. The overall map of pathway deregulation suggests that dynamic changes in specific pathways vary between patients. To examine this pathway‐specific divergence in dynamics, we plotted PDS of matched samples for each patient (Fig. 1E and Fig. S4).

We observed that, while in some patients, pathways returned to normal scores after treatment, in other patients pathways remained at the deregulated state. For example, the Fanconi Anemia pathway is highly deregulated in the TNBC patients and remains deregulated also posttreatment, but in patients ST‐7 and ST‐1, PDS returns to normal levels posttreatment. PDS scores provide a global view of the entire pathway deregulation; however, the routes to adaptive resistance may vary among pathways and patients. Thus, to understand whether the variability among patients in the same pathway is attributed to either specific genes or diverse genes, we proceeded to analysis of temporal modulations at the gene level per‐patient.

### 3.3 Longitudinal pattern classification to identify gene expression patterns associated with response

**Fig. 2:**
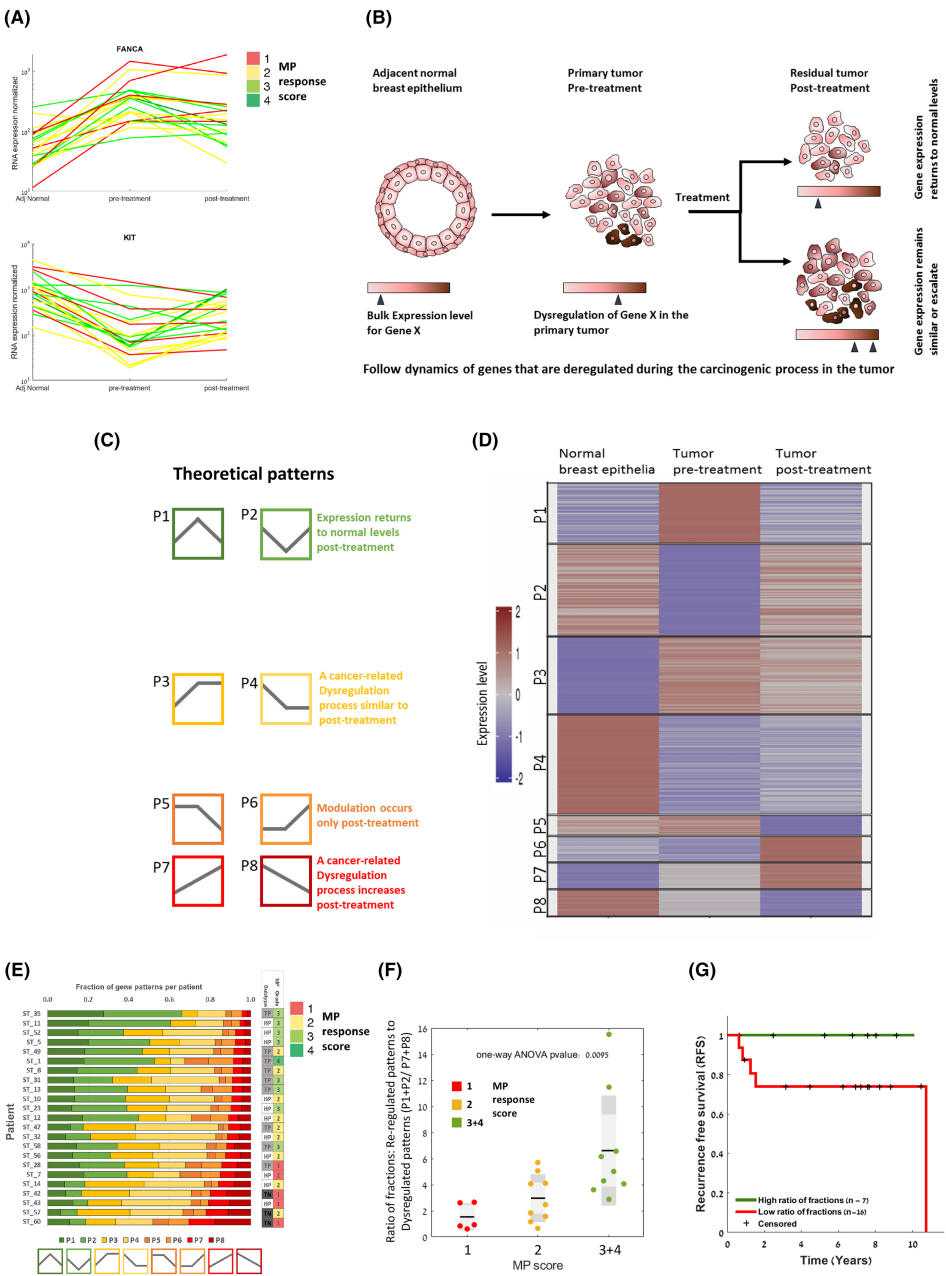
Longitudinal pattern classification to identify gene expression patterns associated with response. (A) Most genes exhibited divergent expression dynamics across patients (*n* = 23) between timepoints, with patterns typically associated with treatment response, as shown in the line graph. (B) A scheme presenting the hypothesis that divergent dynamics in bulk expression can pinpoint genes that are associated with resistance to treatment. (C) Differentially expressed genes were classified into eight possible theoretical patterns of modulations. The various patterns and their suggested scenarios are presented in different colors. (D) Heatmap of all gene‐per‐patient triplets after classification by correlating the Variance Stabilizing Transformation values to the predefined theoretical patterns. Each row corresponds to one gene in a certain patient. The same gene is plotted for each patient, according to its classified pattern in an individual patient. (E) Fractions of genes in the various patterns for each patient. Patients are ordered by the percentage of genes with patterns P7 and P8, which represent increased dysregulation posttreatment relative to the normal levels. (F) Boxplot showing the distribution of the ratio of “reregulated patterns” to “dysregulated patterns” (P1 + P2/P7 + P8), across three MP score groups (*n* = 23 patients) (black line – mean; light gray box – Std; dark gray box – 95% SEM). This ratio was significantly associated with MP response score (one‐way ANOVA, *P*‐value = 0.0095). (G) Kaplan–Meier curve of recurrence‐free survival for the entire cohort (*n* = 23 patients) by their ratio of fractions: (P1 + P2/P7 + P8), cutoff = 5, log rank *P*‐value 0.09. MP, Miller–Payne response score.

Inspecting the temporal gene modifications, we observed that the dynamics of gene expression, rather than its absolute level, is predominantly patient‐specific. Typically, genes showed divergent expression dynamics that were response‐related (Fig. 2A and Fig. S5) with the exception of a few genes showing patient‐wide patterns (Table S2; Fig. S6A). These few genes with patient‐wide patterns were downregulated in the primary tumors relative to adjacent normal tissue, as validated in the TCGA datasets, indicating that this difference is not due to treatment effects in the adjacent normal tissue (Fig. S6B). These few genes typically show highest coexpression of gene‐pairs among patients in single‐timepoint datasets (TCGA and METABRIC, Table S3 and Fig. S6C), indicating their mutual regulation.

For most genes, the temporal patterns, rather than the absolute expression values, were correlated with a treatment response score. For example, the absolute pretreatment expression levels of *FANCA* or *KIT* (Fig. 2A and Fig. S5) did not separate between the various pathological MP response groups. Rather, the dynamic expression pattern differentiated between response groups (Fig. 2A and Fig. S5). This divergence in dynamics led us to hypothesize that classifying genes by their expression pattern can identify resistance‐related genes, as depicted in a scheme in Fig. 2B. We thus performed dynamic pattern analysis for the differentially expressed genes, similar to our previous studies [28, 29], exploiting the strength of the longitudinal dataset. We defined eight possible theoretical patterns (P1–P8) of gene expression modulations through the matched triplet of normal‐tumor‐treatment stages (Fig. 2C). Each pattern represents a different scenario of events through tumor progression and treatment stages:

1. Increased (P1) or decreased (P2) expression levels from normal to pretreated sample and return to normal levels posttreatment. This may represent reregulation of genes posttreatment.
2. Increased (P3) or decreased (P4) expression levels from normal to a pretreated sample, which remain similar to posttreatment, possibly representing a resistance state.
3. Similar expression between normal and tumor samples, which decrease (P5), or increase (P6) only after posttreatment, that may be inferred as treatment effects.
4. Constant increase (P7) or constant decrease (P8) in expression levels from normal through a pretreated sample to a posttreatment sample. This may represent a worsening of the cancerous process.

Genes‐per‐patient trajectories were classified into the various patterns by correlating the Variance Stabilizing Transformation (VST) values (examples of theoretical patterns and assigned genes in Fig. S7). A heatmap presenting the distribution across patterns of all gene‐per‐patient trajectories is shown in Fig. 2D. The same gene is plotted for all patients, according to its patient‐specific classified pattern. To validate the classified pattern directions, we compared the fold changes between tumor‐normal pairs to fold changes observed in the METABRIC datasets (fold change of 1.5 and false discovery rate [FDR] ≤ 0.05) [8] and found an agreement in the direction of upregulation or downregulation in tumor vs normal tissue.

### 3.4 Longitudinal gene pattern fractions are associated with response

To examine the association between expression patterns and response, we plotted the fraction of genes assigned to each pattern per‐patient (Fig. 2E). Patients with a better response score (higher MP value) showed higher fractions of genes with patterns P1 and P2, in which expression returns to normal levels. Poor responders showed higher fractions of P3 or P4 and mainly of P7 or P8, suggesting an increase in cancer‐related processes posttreatment. Notably, cellularity of cancer cells in the sample was not associated with the fraction of patterns. We found that the ratio between the fractions of genes with patterns P1 and P2 relative to P7 and P8 (P1 + P2/P7 + P8), was significantly associated with MP response score (analysis of variance [ANOVA] test, *P*‐value = 0.0095) (Fig. 2F). This fraction of patterns was also associated with recurrence‐free survival, although not significant due to the small cohort size (Fig. 2G). The MP score for this cohort was also associated with recurrence‐free survival (Fig. S8, ns), emphasizing its clinical validity [31]. Altogether, the data suggest that a higher fraction of genes with reregulated patterns is associated with better response and a higher fraction of genes with dysregulated patterns is associated with poor response. These findings motivated the analysis of expression patterns for identifying genes associated with resistance.

### 3.5 Gene patterns analysis identifies genes and pathways associated with response that converge into confined resistance modules

**Fig. 3:**
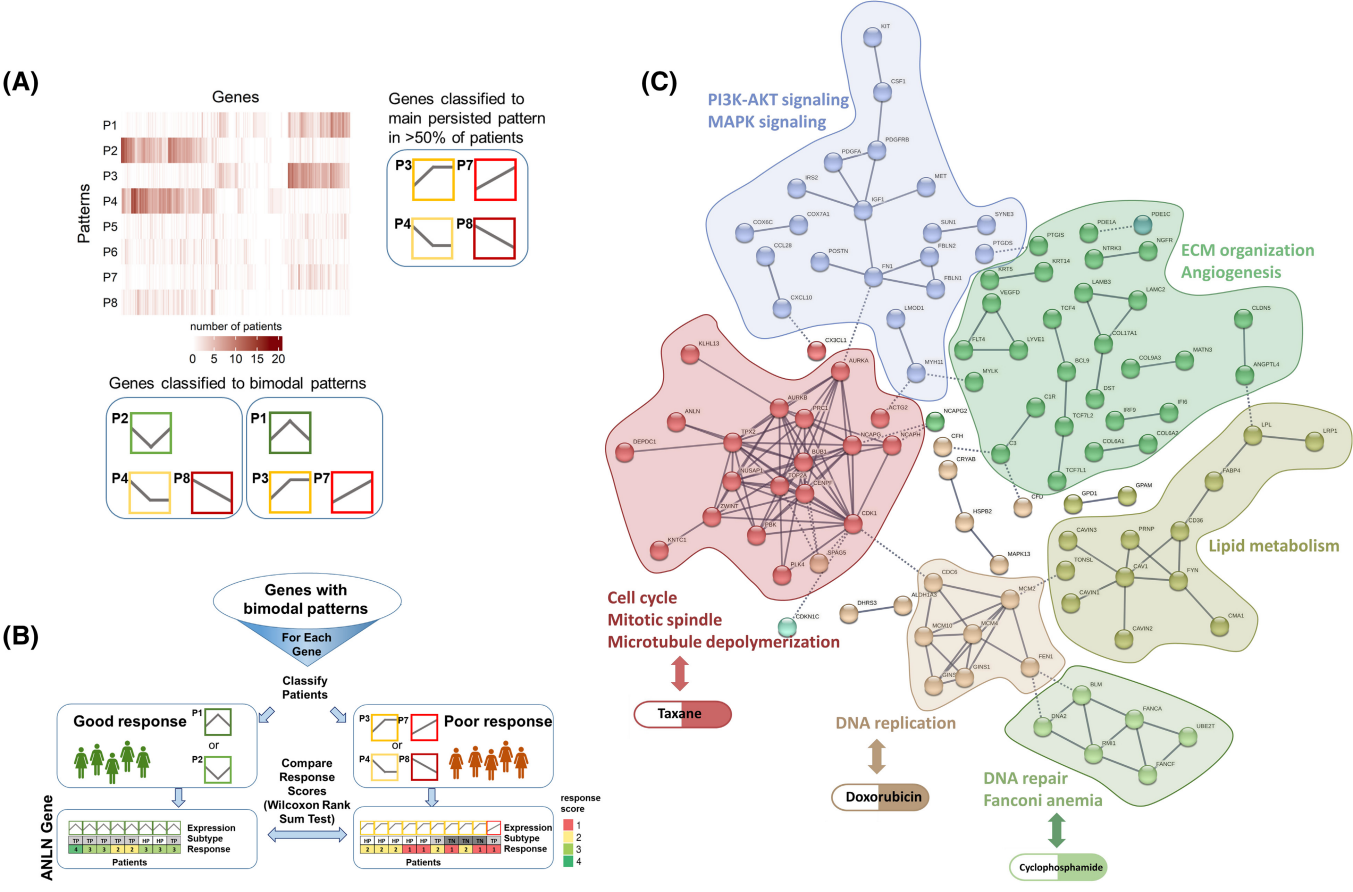
Gene patterns analysis identify genes and pathways associated with resistance that converge into confined resistance modules. (A) Flow chart of the gene pattern analysis algorithm: Differentially expressed genes classified to dynamic patterns typically exhibit one main pattern or bimodal patterns, (for example, P1 or P3). (B) For genes with bimodal patterns, we compared the MP pathological response score of the patients (using the Wilcoxon Rank sum test). We identified 254 genes where their pattern is significantly different between good responders and poor responders. (C) Network analysis (STRING) for all 452 genes associated with resistance (198 main persisted patterns and 254 genes differentiating good and bad responders) demonstrates convergence of the resistance genes into a confined number of resistance modules. Importantly, three modules are strongly associated with the mechanism of action of the administered chemotherapies.

We next sought to pinpoint genes that may have a role in adaptive resistance by searching genes with recurrent dysregulated patterns across patients. To understand how the various patterns are distributed among patients, we clustered the matrix of number of patients per pattern for all differentially expressed genes (~6900 genes). We observed that, frequently, genes were classified into either one main pattern or to bimodal patterns across patients (Fig. 3A). We hypothesized that a gene can be associated with adaptive resistance if its main pattern across most patients (>50%) is one of the dysregulated patterns P3/P7 or P4/P8, namely, that the expression of a cancer‐related gene persists or increases after treatment. We found 198 genes with persistent dysregulated patterns that recur across patients (Table S4). Alternatively, genes associated with resistance can also exhibit bimodal patterns across patients, for which the same gene can be dysregulated in one patient and reregulated in another patient. We speculated that this bimodal distribution among patients could differentiate between good responders and poor responders (Fig. 3B). We thus compared between the MP response score of good responders and poor responders for genes with bimodal patterns using the Wilcoxon Rank sum test and identified 254 significant genes (Fig. 3B) (Table S5). To account for subtype bias, we repeated this test for HR+ patients only (20 patients) and found 122 significant genes (Table S5).

To assess whether treatment affects adjacent normal samples, we compared the expression levels of 452 resistance‐associated genes across normal samples (both adjacent and healthy) and posttreatment tumors. Our analysis revealed that adjacent normal breast tissue from cancer patient clusters with healthy normal breast tissue distinctly separate from posttreatment tumors. This clustering pattern indicates minimal treatment effects on adjacent normal tissues (Fig. S9A). We further emphasized this finding by focusing on a patient with bilateral breast cancer (ST‐52), where the separation between adjacent normal and posttreatment tumor samples is particularly evident (Fig. S9B). To understand the functional connectivity between the identified resistance‐related genes (total of 452), we performed STRING network analysis. We found that the genes converge to a finite number of resistance modules (Fig. 3C). Notably, three modules were related to the mechanism of action of the administered chemotherapies (taxane, cyclophosphamide and doxorubicin) [48]. Taxanes act by disrupting microtubule function, thereby inhibiting mitosis; doxorubicin targets DNA replication and cyclophosphamide exerts its cytotoxic effects mainly by cross‐linking DNA strands. We identified seven genes that were enriched in the DNA repair pathway, mainly in the Fanconi Anemia pathway (Table S6). Other emerging resistance modules were related to extracellular matrix (ECM) remodeling, PI3K‐AKT and MAPK signaling, angiogenesis regulation, glucose transport, and lipid metabolism (Fig. 3C).

To further explore the functional context of the resistance‐associated genes, we performed gene‐set enrichment analysis by clusterprofiler [45] using the combined datasets (GO_biological process KEGG and Reactome) downloaded from EnrichR [44]. We calculated the enrichment separately for each set of bimodal patterns (P1_P3_P7 and P2_P4_P8) and identified 152 pathways significantly associated with resistance (*q* value < 0.05) (see Table S6). We then repeated the enrichment for resistance‐associated genes that were significant for HR+ patients, identifying 78 enriched pathways (*q* value < 0.05) (Table S6). To end up with a robust list of enriched pathways, accounting for subtype heterogeneity, we used the intersection between the two analyses. Pathways were related to cell cycle, mitosis, DNA replication and DNA repair as well as to MAPK and PI3K cascade, glucose transport, lipid transport, and extracellular matrix organization.

### 3.6 Individual patients exhibit distinct dysregulation of diverse genes within pathways

**Fig. 4:**
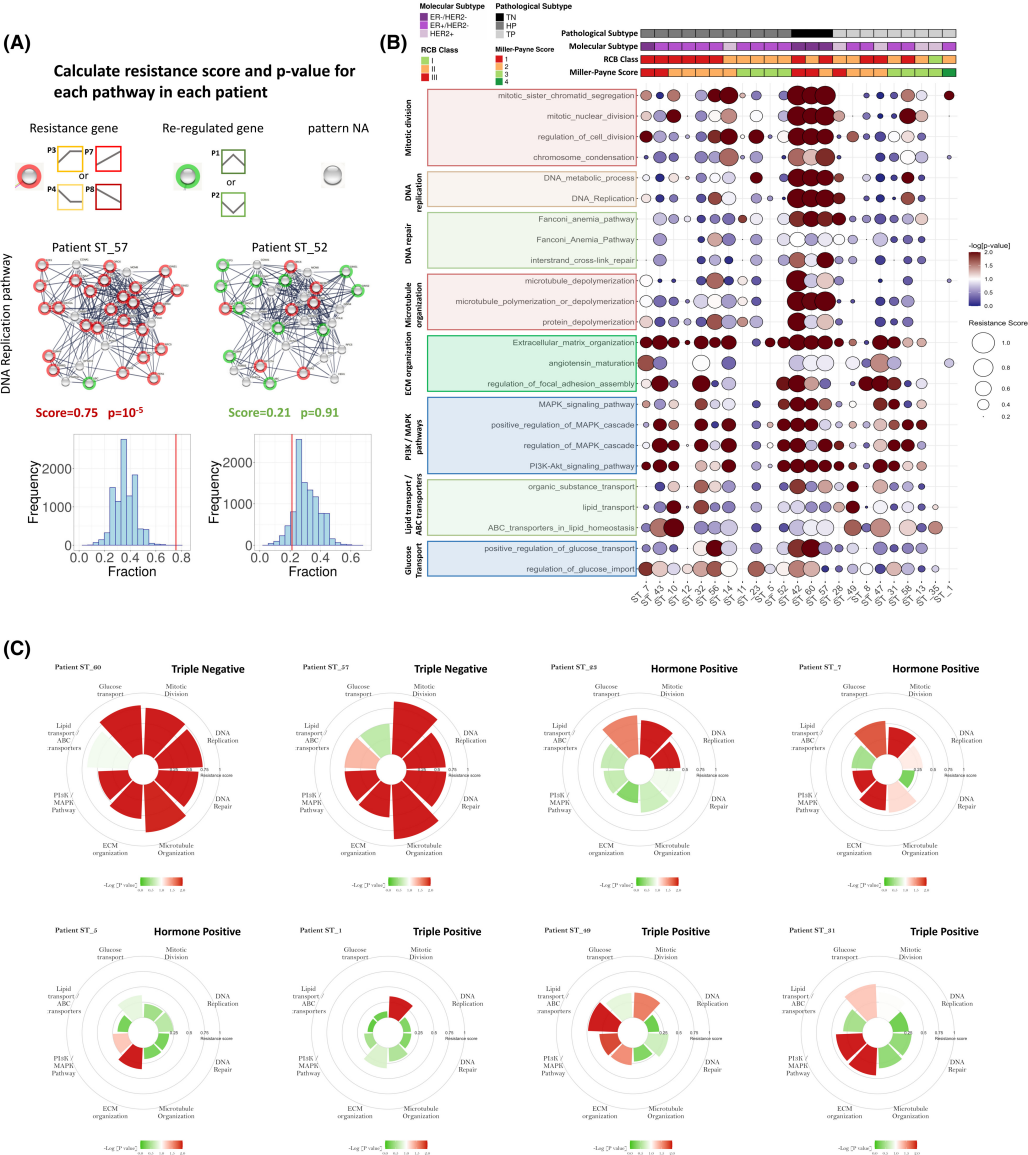
Pathway resistance scores and individual chemoresistome maps demonstrate divergence across patients. (A) For each resistance‐associated pathway in each patient, genes were assigned a mode of either resistance or reregulation based on their classified pattern. We then calculated a resistance score: fraction of “resistant genes” in the pathway and a *P*‐value for this score, calculated by bootstrapping a random set of genes in the same size of the pathway (sampled 10,000 times). An example for the calculated resistance score in two representative patients for the DNA replication pathway, presented as a network. Red dots: resistance genes, green dots: reregulated genes, gray dots: genes with no assigned pattern. The *P*‐value calculation is presented as a histogram of the distribution of random fractions in this patient. The red line denotes the resistance score (fraction of resistant genes in this pathway for this patient). (B) Resistance score and *P*‐value for 24 representative pathways enriched for resistance‐related genes presented as a balloon plot. Each row represents a pathway and each column represents a patient. Circle size denotes the resistance score and color‐code denotes the *P*‐value of the resistance score. Clinical parameters for each patient are color‐coded in the upper panel. The pathways are gathered according to their functional module, in the same color as in the STRING network in A. (C) Personal chemoresistome maps for representative patients exemplifying the divergence in adaptive resistance for each patient. The maps represent the main resistance categories, calculated for the most significant pathways in each category. The resistance score is denoted by the bar height and the *P*‐value is color‐coded. HP, hormone positive; RCB, residual cancer burden; TN, triple‐negative; TP, triple‐positive.

To understand the divergence in dysregulated genes between the patients, we focused on pathways that differentiated good and bad responders and inspected all expressed genes in the dysregulated pathways, for each individual patient. We defined genes with patterns P1/P2 as “reregulated genes” and genes with patterns P3/P4/P7/P8 as “resistance genes.” We observed that the fraction and identity of resistance genes in the same pathway varied between patients. We therefore calculated for each patient a resistance score— the fraction of resistance genes per pathway. The significance of this score was calculated by bootstrapping random sets of genes in the same size of the pathway in each patient (sampled 10 000 times). Calculation of the resistance score for two patients is exemplified by a network map of a representative pathway (Reactome: DNA Replication), and the *P*‐value is represented by histograms of the random fractions of resistance genes for this pathway (Fig. 4A).

The resistance score and *P*‐value per‐patient for a curated list of 24 significant resistance‐associated pathways is presented by a balloon plot, demonstrating the variability in dysregulated pathways between patients (Fig. 4B and Table S7). Importantly, it is evident from the plot that in some patients multiple pathways account for resistance, while in other patients only a few pathways exhibit high resistance scores. A clear difference between subtypes is evident in TNBC patients (ST_42, ST_57, and ST_60), exhibiting multiple resistance‐related pathways typically associated with cell division, while in HR+ patients, resistance‐related pathways vary and are specific to each patient. Although the variation between subtypes is marked, interpatient variability is evident also between the patients harboring the same subtypes and receiving the same treatments. For example, patient ST‐11 and patient ST‐35 show a significant resistance score in only a few pathways, i.e., the Fanconi Anemia pathway in ST‐11 and the ABC transporters pathway in ST‐35 (Fig. 4B). In contrast, patients ST‐10, ST‐32, and ST‐7 exhibit significant resistance scores in multiple pathways. Although only three patients had TNBC, the inclusion of this subtype in the cohort enabled pinpointing pathways that are less represented in HR+ but were evident in several HR+ patients, such as mitotic division in ST‐1, microtubule organization in ST‐32, and DNA‐repair in ST‐8 (Fig. 4B). This divergence in specific dysregulated pathways in response to the same chemotherapies pinpoint the individual emergence of adaptive resistance.

To investigate potential associations between emerging resistance in DNA repair pathways and specific mutations linked to homologous recombination deficiency, we conducted genetic testing using a panel of 50 mutations. This panel included 30 BRCA1/2 mutations, selected to cover the most prevalent variants in our study population based on ethnicity. Among the 22/23 patients tested, 20 were negative for all screened mutations. Three patients showed positive results: ST‐1 and ST‐28 harbored CHEK2 mutations, while ST‐52 carried a BRCA2 mutation (Table S1). Notably, significant resistance scores for the Fanconi Anemia pathway or interstrand crosslink repair pathways were observed in patients ST‐11, ST‐42, ST‐57, and ST‐28. Our analysis didn't find a clear association between the identified genetic mutations and elevated resistance scores in these pathways. These findings suggest that the observed adaptive resistance in DNA repair pathways may not be directly attributable to preexisting mutations in the genes we tested.

To highlight the distinct contribution of each module to the emergence of resistance in individual patients, we generated personal chemoresistome maps by circular bar‐graphs (Fig. 4C). The maps exemplify the coexistence of several resistance modules/mechanisms in each patient, while highlighting interpatient variability by the impact of each module. Importantly, some modules exhibited a patient‐specific or subtype‐specific occurrence, emphasizing the individual emergence of resistance. We observed that resistance modules related to cell cycle and cell division are abundant in TNBC and fast‐dividing tumors, while other modules, such as ECM remodeling and PI3K/AKT regulation, were more characteristic of HR+ tumors. Nevertheless, cell proliferation modules were also evident in non‐TNBC patients, but are frequently more sporadic and specific to a mechanism‐of‐action of one drug.

### 3.7 Variability in gene and pathway dysregulation reveals the principles in adaptive chemo‐resistance

**Fig. 5:**
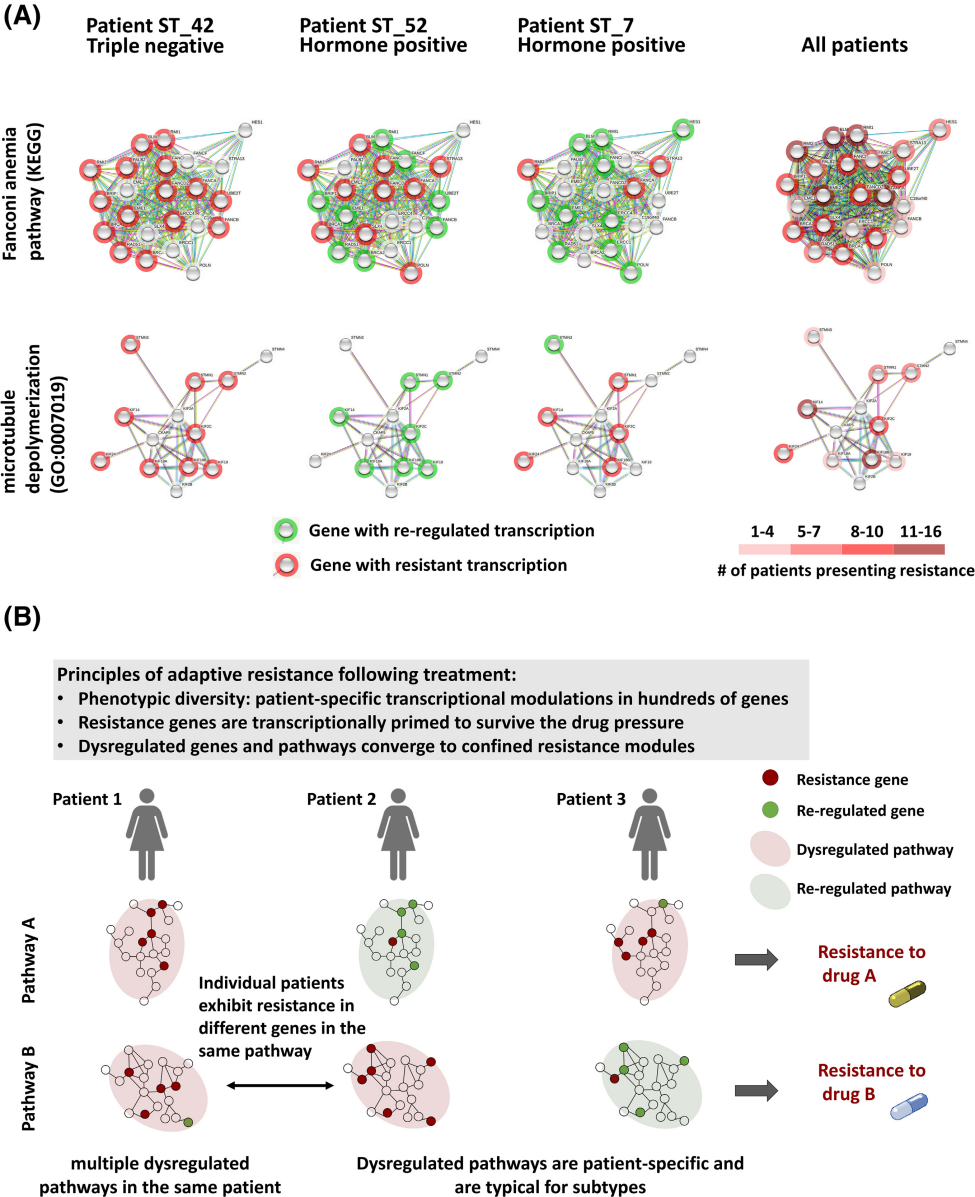
Variability in gene rewiring and pathway dysregulation reveals the principles of adaptive resistance following chemotherapy. (A) An example for the variability in pathway dysregulation between patients. Network presentation of the expressed genes in two representative pathways, colored by the gene pattern analysis to classify genes into either reregulated or resistance genes (see Fig. 4B). While patient ST_42 (TNBC) exhibited multiple resistance genes in both pathways, patient ST_7 and patient ST_52 exhibited an opposite resistance tendency for the two pathways. On the right: the same pathways, represented as a heatmap for the number of patients with resistance pattern per gene, demonstrating gene hubs for resistance in the pathway. (B) Principles of adaptive resistance following chemotherapy. Hundreds of genes undergo rewiring following chemotherapy, in a patient‐specific manner. The resistance‐related genes are frequently transcriptionally primed in the primary tumor. The same pathway can undergo dysregulation by rewiring different genes. The rewired genes and dysregulated pathways converge into confined resistance modules, typically related to the mechanism of action of the administered drugs.

To further explore the interpatient variability within dysregulated pathways, we zoomed into the various genes in each resistance‐associated pathway. When comparing the resistance genes between patients, we found that there are hubs of resistance genes (Patterns P3, P4, P7 and P8), that recur in many patients. These hubs are represented as a heatmap for the number of patients with resistance pattern per gene (Fig. 5A: right panel and Fig. S10).

The interpatient variability of the rewired genes is exemplified for two pathways by gene network maps (Fig. 5A). For example, in the Fanconi Anemia pathway, many genes exhibited resistance patterns in patient ST_42 (TNBC), while in patients ST_7 (HR+) and ST_52 (HR+) the number of resistance genes varied (Fig. 5A). Specifically, the genes in this pathway for patient ST_7 exhibit mostly reregulated patterns, although all patients received cyclophosphamide. The intervariability between these patients was also evident in the microtubule depolymerization pathway. For example, in the Fanconi Anemia pathway, RMI2 and FANCA demonstrated resistance patterns in 13 patients (Fig. S11A,B). Importantly, patient ST_52 did not receive paclitaxel and we identified only reregulated genes in the microtubule depolymerization pathway (Fig. S11C), suggesting that resistance‐related rewiring is induced by Taxane, that targets this pathway. However, the variability between patients is high and we observed only reregulated genes in other patients (mostly HR+/HER2+) who received paclitaxel (Fig. S11C).

Altogether, the results of this study led us to suggest the principles of adaptive resistance postchemotherapy (illustrated in Fig. 5B). We propose that hundreds of genes undergo rewiring following chemotherapy. The rewired genes are mostly cancer‐related genes that were already modulated during the carcinogenic process relative to the normal epithelium tissue, some of which return to normal levels, while others maintain dysregulated levels. Although many genes and pathways exhibit dysregulation posttreatment, they converge to a finite number of confined resistance modules. Furthermore, rewired genes and dysregulated pathways are patient‐specific; thus, different genes in the same pathway can be rewired in individual patients.

## 4 Discussion

The results of this study provide a wide perspective on the complexity of cancer cells adaption to chemotherapies. Our study design and analysis approach differ from previous studies in two ways: (a) Examining alterations in relation to the corresponding normal epithelium; (b) Deconstructing pathways to dynamic pattern modulation for each gene per patient, thereby exposing the multiple routes to emergence of an adaptive resistance in individual patients. Inspecting patient‐specific modulations before and after treatment relative to the normal epithelium of the same patient revealed that rewiring of gene expression is a composite interplay, unique to each tumor. We postulate that multiple pathways, already dysregulated in the primary tumor, maintain a drug‐tolerant state by activating many genes that safeguard their abnormal activity. Thereby, to survive the toxic activity of a drug, tumor cells either sustain the dysregulated state or intensify it, specifically bypassing the drug's interference.

Our approach of longitudinal sampling, combined with pattern analysis, emphasized the importance of inspecting dynamic changes to reveal mechanisms of drug resistance. Most resistance genes could not be detected in a single timepoint analysis, as their absolute levels in the primary tumor were indistinguishable between good and poor responders. For example, the TOP2A gene, specifically targeted by doxorubicin, was previously evaluated as a marker for benefit from this drug [49], but inconsistent results impaired its predictive efficacy. Our data clearly indicate that TOP2A expression levels in the primary tumor are inconsistent with response, but its longitudinal expression pattern differentiates between good and poor responders (Fig. S5). This may explain why heterogeneous tumor phenotypes challenge the identification of molecular markers and suggests that patient‐specific dynamic changes are crucial to target the resistance genes and pathways.

A key advantage of our dataset is the inclusion of matched adjacent normal tissue. In previous longitudinal studies, the search for differentially expressed genes before and after treatment missed nonmodified genes that sustain a dysregulated state, as in Pattern P3. On the other hand, genes that are frequently modified posttreatment but are actually reregulated (patterns P1/P2), can be misinterpreted as markers for chemoresistance (exemplified in a scheme, Fig. S12). For example, CYR61 was previously reported to be upregulated post‐NAT [23], and was suggested as a chemoresistance marker. In our dataset, its levels are indeed upregulated post‐NAT, but to similar levels as in adjacent normal tissue, thus inferred as a reregulated gene (Fig. S12A). In contrast, upregulation of FN1 post‐NAT was previously associated with poor response [50] and mainly exhibited resistance patterns in our dataset (Fig. S12B). Importantly, adjacent normal tissues may be affected by surrounding carcinogenic processes, a phenomenon known as field cancerization, or may be predisposed to cancer due to genetic factors. Therefore, it is crucial to validate that adjacent normal tissues are similar to healthy normal breast tissue, as demonstrated in Fig. S3. However, in longitudinal studies comparing each tumor to its own adjacent normal is the most relevant reference. Similarly, it is essential to confirm that the changes observed in posttreatment tumors are specific to the cancer tissue and do not globally affect the adjacent normal tissue, as illustrated in Fig. S9.

The fact that most resistance genes were identified in patterns P3/P7 or P4/P8, modulated in the carcinogenic state relative to the normal epithelium, suggests that tumors resist treatment by maintaining dysregulated pathways. Signaling pathways that are crucial for cancer cells survival sustain their activity or shift to higher gears to persist under chemotherapy extreme conditions. Our observations suggest that persistent subpopulations possess primed transcriptional profiles, relative to the normal state, that convey gene expression bias towards survival. This notion is in accordance with recent *in vitro* studies of drug‐tolerant cells, showing that phenotypic resistance is determined *a priori* in a nonstochastic manner in subpopulations of persister cells [51, 52]. Another study of single‐cell sequencing of TNBC patients in response to chemotherapy supports this concept, showing clonal selection of preexisting genotypes, as well as transcriptional reprogramming of resistant signatures in the persistent clones [6]. Similar to our results, they found that transcriptional programs converged to a few resistance pathways and were mostly acquired after treatment. Our data confirm the concept that resistant phenotypes were already acquired in the primary tumor, relative to matched normal tissue, and their dysregulation had either persisted or intensified. However, it is important to emphasize that although these genes are primed in the primary tumor, in many patients their expression is normalized, thus profiling the primary tumor is not sufficient to determine these genes or their downstream pathway as a potential resistant mechanism.

Dynamic changes in response to chemotherapy may occur as a result of genetic selection or by massive transcriptional adaptation. The kinetics of transcriptional adaptation are considerably faster than genetic evolution, and it is assumed that transient nongenetic mechanisms will eventually be hardwired through long‐term epigenetic or genetic mechanisms [2]. *In vitro* pattern analysis of time‐course omics during treatment revealed immediate massive transcriptional adaptation followed by concordant epigenetic alterations, suggested to stabilize the resistant phenotype [53]. Notably, our study measures modulation in the bulk expression levels that can represent either transcriptional selection of cells expressing a gene or a transcriptional adaptation by gene rewiring. Either way, the net levels point towards the dysregulation or reregulation state of the tumor. As shown in our study and in previous studies [54], gene rewiring converges to a confined set of drug‐resistant phenotypes. However, while most studies focus on genes and pathways that are shared between patients, we emphasize the phenotypic heterogeneity between patients and the divergent routes of adaptive resistance.

The emergence of key signaling pathways in our dataset, directly related to the mechanism of action of the chemotherapies, resembles the mechanism of resistance to kinase inhibitors, by which persistent activation of the drug target bypasses its inhibitory effect [1]. Nevertheless, the results also indicate an acquisition of an additional resistance mechanisms, such as angiogenesis, extracellular matrix (ECM)‐related, PI3K/AKT signaling, and lipid metabolism alterations. While in TNBC tumors proliferation pathways are mainly upregulated (P1), in HR+ tumors differentiation pathways are downregulated (P2). In TNBC tumors dominant resistance pathways were related to cell cycle, mitotic spindle, the Hedgehog pathway, DNA replication, and DNA repair, indicative of these highly proliferative tumors. In HR+ tumors, we mainly observed resistance patterns in PI3K/Akt pathways, regulation of the MAPK cascade, ECM‐related pathways, lipid transport, and ABC transporter pathways. Indeed, nongenomic activity of estrogens has been suggested to induce chemoresistance, activating PI3K/AKT, and modifying DNA damage response [55]. Resistance patterns emerged in specific subsets of ABC transporters, the ABCA2 family, involved in steroid transport that may regulate breast cancer proliferation and survival, as was suggested in a prostate cancer study [56].

Our findings may have several future clinical implications. Overcoming the drug‐tolerant state in the clinical setting is challenging, given that there is no one driver mutation to target. Our data suggest that while many resistant phenotypes are emerging in parallel, reregulated pathways disclose their sensitivity, enabling us to rationally select the next therapeutic plan. Residual tumors after NAT have a higher incidence rate for recurrence [15]. These residual tumors harbor distinct molecular profiles that disclose their acquired resistance on one the hand, and their vulnerabilities (i.e., reregulated genes upon treatment) on the other hand. Therefore, profiling the chemoresistome immediately after surgery may guide the adjuvant treatment plan of targeted therapy to prevent recurrent or metastatic disease. Although profiling pre‐ and posttreatment biopsies in clinical practice is challenging, future developments such as cell‐free RNA or epigenetic profiling of tumor DNA can lead to chemoresistome profiling during the initial cycles of the neoadjuvant treatment. Moreover, recent advances make it possible to evaluate various treatments *ex vivo* on tumor biopsies adjacent to diagnosis [57]. Chemoresistome mapping based on gene expression patterns of tumor samples treated *ex vivo* prior to NAT can serve to optimize personalized treatment plans. *Ex vivo* treatment allows estimation of resistance to both single agents and drug combinations. For cost‐effectiveness, profiling the chemoresistome genes rather than the entire transcriptome may be beneficial, though it may limit the breadth of information. For instance, identifying a high resistance score in microtubule depolymerization pathways may indicate potential adaptive resistance for taxanes, while a high resistance score in Fanconi Anemia genes may indicate emergence of adaptive resistance to cyclophosphamide. This approach can be extended to create resistome maps for immunotherapy (immunoresistome) or other targeted therapies, enabling more precise treatment selection.

The results of our study are limited by the small cohort size and the diverged subtypes. The findings and suggested principles should be further validated in larger cohorts, as well as in other treatment regimens. While the cohort size may limit generalizability, the longitudinal framework provides valuable insights into patient‐specific resistance mechanisms, which is a crucial step towards understanding and potentially overcoming adaptive resistance in a personalized medicine context. Although the cohort size was too small for subgroup analysis, we claim that the combined subtypes analysis was advantageous for detecting patient‐specific modulations. Many resistance‐related modulations that were subtype‐specific, mainly in TNBC, were found in few patients from other subtypes. In addition, the study focused on NAT, where systemic treatment is given in the presence of the primary tumor. It is difficult to determine whether the concepts of adaptive resistance, as derived from the NAT dataset, apply to adjuvant treatment settings, in which no clinically‐evident tumor is present. Lastly, although we enriched our samples for epithelial cell by macrodissection, confounding factors that may impair the inference of our analysis are related to stroma‐associated changes after treatment, including overrepresentation of collagen and immune cells. A significant limitation of bulk tumor RNA‐seq data is that it represents a mixture of cell types. While our gene expression data clustered according to sample type and was not overtly affected by tumor cellularity, we cannot entirely rule out the possibility that some observed gene expression patterns reflect modulations in other cell types or changes in their proportions posttreatment. Moreover, another limitation is the potential overrepresentation of dominant subclones from the sampled spatial domain, particularly posttreatment surviving subclones. However, as we focused on recurrent and shared patterns across patients to identify chemoresistome pathways, we believe that any potential bias due to tumor heterogeneity had minimal impact on our results. Nevertheless, future single‐cell experiments or spatial transcriptomics assays would be valuable to complement and validate our findings.

## 5 Conclusion

In summary, elucidation of principles governing mechanisms of adaptive resistance to chemotherapy has been facilitated by our unique longitudinal dataset coupled with gene‐pattern classification analysis. A complex and heterogeneous phenotypic adaptation depicted from this study appears as a major obstacle for successful anticancer treatment. Nevertheless, the insightful concepts derived from the study may accelerate development of clinically available methods to estimate the emerged adaptive states, eventually leading to optimization of patient‐specific treatment plans in the future.

## Conflict of interest

ENG‐Y reports Honoraria and Consulting fees from Pfizer, Novartis, and Roche Eli‐Lilly AstraZeneca. No other potential competing interest are relevant to this article are reported.

## Author contributions

MD designed the study concept, supervised the study, achieved funding, performed bioinformatics and statistical analysis, created visualization, and wrote the article. GF performed bioinformatics and statistical analysis, created visualization, and edited the article. GP collected the biopsies and the clinical data, processed the samples, and edited the article. NB‐L performed pathological evaluation. SG designed the mRNAseq protocols and supervised the transcriptomics sequencing. DM‐S quantified the immunohistochemistry staining. AS collected the samples and edited the article. NBB‐M was involved in initial bioinformatics analysis and data quality control. AP processed the samples. RB‐M supervised the oncogenetic tests. ED was involved in data analysis conceptualization and data interpretation. IB supervised the pathological evaluation. TG was involved in study conceptualization, achieved funding, and edited the article. BK (deceased) was involved in study conceptualization and achieved funding. ENG‐Y was involved in data interpretation, provided resources, and edited the article.

## Supporting information


**Fig. S1.** Flow chart of patients and samples used in the analysis.
**Fig. S2.** Correlation between expression levels and scores of pathological markers.
**Fig. S3.** Similarity between normal breast tissue from healthy individuals and adjacent normal tissue from cancer patients.
**Fig. S4.** Temporal modulations in deregulation scores per‐patient for representative pathways.
**Fig. S5.** Diverged temporal expression patterns associated with resistance
**Fig. S6.** . Genes with shared pattern dynamics across patients
**Fig. S7.** Pattern classification by correlating genes to theorethical patterns.
**Fig. S8.** Kaplan–Meier curve of recurrence free survival for the entire cohort by their MP response score.
**Fig. S9.** Distinct clustering of resistance genes in adjacent normal and posttreatment tumor tissues.
**Fig. S10.** Hubs of resistance genes in selected dysregulated pathways.
**Fig. S11.** Heat maps presenting the modes of resistance/reregulation in two representative dysregulated pathways.
**Fig. S12.** Resolving resistance by pattern analysis of matched three timepoints.

**Table S1.** Cohort clinical data.

**Table S2.** Genes with homogenous dynamics.

**Table S3.** FOS co‐expression.

**Table S4.** Main pattern genes.

**Table S5.** Genes associated with response Wilcoxon test.

**Table S6.** Full list of enriched pathways.

**Table S7.** Selected pathways with resistance scores and *P*‐values.

## Data Availability

The RNA‐seq data have been deposited in the database of Gene Expression Omnibus (GEO) and are accessible through GEO Series accession number GSE217624 (https://www.ncbi.nlm.nih.gov/geo/query/acc.cgi?acc=GSE217624).

## References

[1] Garraway LA , Jänne PA . Circumventing cancer drug resistance in the era of personalized medicine. Cancer Discov. 2012;2:214–226. 22585993 10.1158/2159-8290.CD-12-0012

[2] Salgia R , Kulkarni P . The genetic/nongenetic duality of drug ‘resistance’ in cancer. Trends Cancer. 2018;4:110–118. 10.1016/j.trecan.2018.01.001 29458961 PMC5822736

[3] Marine JC , Dawson SJ , Dawson MA . Non‐genetic mechanisms of therapeutic resistance in cancer. Nat Rev Cancer. 2020;20:743–756. 10.1038/s41568-020-00302-4 33033407

[4] Hong SP , Chan TE , Lombardo Y , Corleone G , Rotmensz N , Bravaccini S , et al. Single‐cell transcriptomics reveals multi‐step adaptations to endocrine therapy. Nat Commun. 2019;10:1–14. 31477698 10.1038/s41467-019-11721-9 PMC6718416

[5] Echeverria GV , Ge Z , Seth S , Zhang X , Jeter‐Jones S , Zhou X , et al. Resistance to neoadjuvant chemotherapy in triple‐negative breast cancer mediated by a reversible drug‐tolerant state. Sci Transl Med. 2019;11:eaav0936. 30996079 10.1126/scitranslmed.aav0936 PMC6541393

[6] Kim C , Gao R , Sei E , Brandt R , Hartman J , Hatschek T , et al. Chemoresistance evolution in triple‐negative breast cancer delineated by single‐cell sequencing. Cell. 2018;173:879–893.e13. 29681456 10.1016/j.cell.2018.03.041 PMC6132060

[7] Harbeck N , Penault‐Llorca F , Cortes J , Gnant M , Houssami N , Poortmans P , et al. Breast cancer. Nat Rev Dis Primers. 2019;5:66. 31548545 10.1038/s41572-019-0111-2

[8] Curtis C , Shah SP , Chin S‐F , Turashvili G , Rueda OM , Dunning MJ , et al. The genomic and transcriptomic architecture of 2,000 breast tumours reveals novel subgroups. Nature. 2012;486(7403):346–352. 10.1038/nature10983 22522925 PMC3440846

[9] Shah SP , Roth A , Goya R , Oloumi A , Ha G , Zhao Y , et al. The clonal and mutational evolution spectrum of primary triple‐negative breast cancers. Nature. 2012;486:395–399. 22495314 10.1038/nature10933 PMC3863681

[10] TCGA . Comprehensive molecular portraits of human breast tumours. Nature. 2012;490:61–70. 23000897 10.1038/nature11412 PMC3465532

[11] Stephens PJ , Tarpey PS , Davies H , Van Loo P , Greenman C , Wedge DC , et al. The landscape of cancer genes and mutational processes in breast cancer. Nature. 2012;486(7403):400–404. 10.1038/nature11017 22722201 PMC3428862

[12] Banerji S , Cibulskis K , Rangel‐Escareno C , Brown KK , Carter SL , Frederick AM , et al. Sequence analysis of mutations and translocations across breast cancer subtypes. Nature. 2012;486:405–409. 22722202 10.1038/nature11154 PMC4148686

[13] Drago JZ , Modi S , Chandarlapaty S . Unlocking the potential of antibody–drug conjugates for cancer therapy. Nat Rev Clin Oncol. 2021;18:327–344. 10.1038/s41571-021-00470-8 33558752 PMC8287784

[14] Selli C , Sims AH . Neoadjuvant therapy for breast cancer as a model for translational research. Breast Cancer. 2019;13:1178223419829072. 30814840 10.1177/1178223419829072 PMC6381436

[15] Symmans WF , Wei C , Gould R , Yu X , Zhang Y , Liu M , et al. Long‐term prognostic risk after neoadjuvant chemotherapy associated with residual cancer burden and breast cancer subtype. J Clin Oncol. 2017;35:1049–1060. 28135148 10.1200/JCO.2015.63.1010 PMC5455352

[16] Ayers M , Symmans WF , Stec J , Damokosh AI , Clark E , Hess K , et al. Gene expression profiles predict complete pathologic response to neoadjuvant paclitaxel and fluorouracil, doxorubicin, and cyclophosphamide chemotherapy in breast cancer. J Clin Oncol. 2004;22:2284–2293. 15136595 10.1200/JCO.2004.05.166

[17] Balko JM , Cook RS , Vaught DB , Kuba MG , Miller TW , Bhola NE , et al. Profiling of residual breast cancers after neoadjuvant chemotherapy identifies DUSP4 deficiency as a mechanism of drug resistance. Nat Med. 2012;18:1052–1059. 22683778 10.1038/nm.2795 PMC3693569

[18] Gonzalez‐Angulo AM , Iwamoto T , Liu S , Chen H , Do KA , Hortobagyi GN , et al. Gene expression, molecular class changes, and pathway analysis after neoadjuvant systemic therapy for breast cancer. Clin Cancer Res. 2012;18:1109–1119. 22235097 10.1158/1078-0432.CCR-11-2762 PMC3288822

[19] Balko JM , Giltnane JM , Wang K , Schwarz LJ , Young CD , Cook RS , et al. Molecular profiling of the residual disease of triple‐negative breast cancers after neoadjuvant chemotherapy identifies actionable therapeutic targets. Cancer Discov. 2014;4:232–245. 24356096 10.1158/2159-8290.CD-13-0286 PMC3946308

[20] Satpathy S , Jaehnig EJ , Krug K , Kim BJ , Saltzman AB , Chan DW , et al. Microscaled proteogenomic methods for precision oncology. Nat Commun. 2020;11(1):532. 10.1038/s41467-020-14381-2 31988290 PMC6985126

[21] Modlich O , Prisack HB , Munnes M , Audretsch W , Bojar H . Immediate gene expression changes after the first course of neoadjuvant chemotherapy in patients with primary breast cancer disease. Clin Cancer Res. 2004;10:6418–6431. 15475428 10.1158/1078-0432.CCR-04-1031

[22] Chang JC , Wooten EC , Tsimelzon A , Hilsenbeck SG , Gutierrez MC , Tham YL , et al. Patterns of resistance and incomplete response to docetaxel by gene expression profiling in breast cancer patients. J Clin Oncol. 2005;23:1169–1177. 15718313 10.1200/JCO.2005.03.156

[23] Magbanua MJM , Wolf DM , Yau C , Davis SE , Crothers J , Au A , et al. Serial expression analysis of breast tumors during neoadjuvant chemotherapy reveals changes in cell cycle and immune pathways associated with recurrence and response. Breast Cancer Res. 2015;17:73. 26021444 10.1186/s13058-015-0582-3 PMC4479083

[24] Silwal‐Pandit L , Nord S , Von Der Lippe Gythfeldt H , Møller EK , Fleischer T , Rødland E , et al. The longitudinal transcriptional response to neoadjuvant chemotherapy with and without bevacizumab in breast cancer. Clin Cancer Res. 2017;23:4662–4670. 28487444 10.1158/1078-0432.CCR-17-0160

[25] Sammut SJ , Crispin‐Ortuzar M , Chin SF , Provenzano E , Bardwell HA , Ma W , et al. Multi‐omic machine learning predictor of breast cancer therapy response. Nature. 2022;601:623–629. 34875674 10.1038/s41586-021-04278-5 PMC8791834

[26] Griffiths JI , Chen J , Cosgrove PA , O'Dea A , Sharma P , Ma C , et al. Serial single‐cell genomics reveals convergent subclonal evolution of resistance as patients with early‐stage breast cancer progress on endocrine plus CDK4/6 therapy. Nat Cancer. 2021;2(6):658–671. 10.1038/s43018-021-00215-7 34712959 PMC8547038

[27] Almendro V , Marusyk A , Polyak K . Cellular heterogeneity and molecular evolution in cancer. Annu Rev Pathol. 2013;8:277–302. 23092187 10.1146/annurev-pathol-020712-163923

[28] Dadiani M , Bossel Ben‐Moshe N , Paluch‐Shimon S , Perry G , Balint N , Marin I , et al. Tumor evolution inferred by patterns of microRNA expression through the course of disease, therapy and recurrence in breast cancer. Clin Cancer Res. 2016;157:157–165. 10.1158/1078-0432.CCR-15-2313 26957561

[29] Shenoy A , Belugali Nataraj N , Perry G , Loayza Puch F , Nagel R , Marin I , et al. Proteomic patterns associated with response to breast cancer neoadjuvant treatment. Mol Syst Biol. 2020;16:1–17. 10.15252/msb.20209443 PMC7507992 32960509

[30] Bossel Ben‐Moshe N , Gilad S , Perry G , Benjamin S , Balint‐Lahat N , Pavlovsky A , et al. mRNA‐seq whole transcriptome profiling of fresh frozen versus archived fixed tissues. BMC Genomics. 2018;19:419. 29848287 10.1186/s12864-018-4761-3 PMC5977534

[31] Ogston KN , Miller ID , Payne S , Hutcheon AW , Sarkar TK , Smith I , et al. A new histological grading system to assess response of breast cancers to primary chemotherapy: prognostic significance and survival. Breast. 2003;12:320–327. 14659147 10.1016/s0960-9776(03)00106-1

[32] Symmans WF , Peintinger F , Hatzis C , Rajan R , Kuerer H , Valero V , et al. Measurement of residual breast cancer burden to predict survival after neoadjuvant chemotherapy. J Clin Oncol. 2007;25:4414–4422. 17785706 10.1200/JCO.2007.10.6823

[33] Martin M . Cutadapt removes adapter sequences from high‐throughput sequencing reads. EMBnet J. 2011;17:10.

[34] Dobin A , Davis CA , Schlesinger F , Drenkow J , Zaleski C , Jha S , et al. STAR: ultrafast universal RNA‐seq aligner. Bioinformatics. 2013;29:15–21. 23104886 10.1093/bioinformatics/bts635 PMC3530905

[35] Anders S , Pyl PT , Huber W . HTSeq—a python framework to work with high‐throughput sequencing data. Bioinformatics. 2015;31:166–169. 25260700 10.1093/bioinformatics/btu638 PMC4287950

[36] Köster J , Rahmann S . Snakemake—a scalable bioinformatics workflow engine. Bioinformatics. 2012;28:2520–2522. 10.1093/bioinformatics/bts480 22908215

[37] R Core Team . R: a language and environment for statistical computing. Vienna, Austria: R Foundation for Statistical Computing; 2021.

[38] Yoshihara K , Shahmoradgoli M , Martínez E , Vegesna R , Kim H , Torres‐Garcia W , et al. Inferring tumour purity and stromal and immune cell admixture from expression data. Nat Commun. 2013;4:2612. 24113773 10.1038/ncomms3612 PMC3826632

[39] Love MI , Huber W , Anders S . Moderated estimation of fold change and dispersion for RNA‐seq data with DESeq2. Genome Biol. 2014;15:550. 25516281 10.1186/s13059-014-0550-8 PMC4302049

[40] Gendoo DMA , Ratanasirigulchai N , Schroder MS , Pare L , Parker JS , Prat A , et al. Genefu: an R/Bioconductor package for computation of gene expression‐based signatures in breast cancer. Bioinformatics. 2016;32:1097–1099. 26607490 10.1093/bioinformatics/btv693 PMC6410906

[41] Wirapati P , Sotiriou C , Kunkel S , Farmer P , Pradervand S , Haibe‐Kains B , et al. Meta‐analysis of gene expression profiles in breast cancer: toward a unified understanding of breast cancer subtyping and prognosis signatures. Breast Cancer Res. 2008;10(4):R65. 10.1186/bcr2124 18662380 PMC2575538

[42] Drier Y , Sheffer M , Domany E . Pathway‐based personalized analysis of cancer. Proc Natl Acad Sci USA. 2013;110:6388–6393. 23547110 10.1073/pnas.1219651110 PMC3631698

[43] Livshits A , Git A , Fuks G , Caldas C , Domany E . Pathway‐based personalized analysis of breast cancer expression data. Mol Oncol. 2015;9:1471–1483. 25963740 10.1016/j.molonc.2015.04.006 PMC5528809

[44] Kuleshov MV , Jones MR , Rouillard AD , Fernandez NF , Duan Q , Wang Z , et al. Enrichr: a comprehensive gene set enrichment analysis web server 2016 update. Nucleic Acids Res. 2016;44:W90–W97. 27141961 10.1093/nar/gkw377 PMC4987924

[45] Yu G , Wang L‐G , Han Y , He Q‐Y . clusterProfiler: an R package for comparing biological themes among gene clusters. OMICS. 2012;16:284–287. 10.1089/omi.2011.0118 22455463 PMC3339379

[46] Szklarczyk D , Gable AL , Lyon D , Junge A , Wyder S , Huerta‐Cepas J , et al. STRING v11: protein‐protein association networks with increased coverage, supporting functional discovery in genome‐wide experimental datasets. Nucleic Acids Res. 2019;47:D607–D613. 30476243 10.1093/nar/gky1131 PMC6323986

[47] Bernstein‐Molho R , Shhada NA , Laitman Y , Netzer I , Shoval S , Friedman E . Targeted genotyping for recurring variants in cancer susceptibility genes in non‐Ashkenazi Jewish patients with breast cancer diagnosed ≥50 years. Cancer. 2024;130:2763–2769. 10.1002/cncr.35329 38630906

[48] Abotaleb M , Kubatka P , Caprnda M , Varghese E , Zolakova B , Zubor P , et al. Chemotherapeutic agents for the treatment of metastatic breast cancer: an update. Biomed Pharmacother. 2018;101:458–477. 29501768 10.1016/j.biopha.2018.02.108

[49] Desmedt C , Di Leo A , De Azambuja E , Larsimont D , Haibe‐Kains B , Selleslags J , et al. Multifactorial approach to predicting resistance to anthracyclines. J Clin Oncol. 2011;29:1578–1586. 21422418 10.1200/JCO.2010.31.2231

[50] Robertson C . The extracellular matrix in breast cancer predicts prognosis through composition, splicing, and crosslinking. Exp Cell Res. 2016;343:73–81. 10.1016/j.yexcr.2015.11.009 26597760

[51] Berger JA , Gigi E , Kupershmidt L , Meir Z , Gavert N , Zwang Y , et al. IRS1 phosphorylation underlies the non‐stochastic probability of cancer cells to persist during EGFR inhibition therapy. Nat Cancer. 2021;2:1055–1070. 35121883 10.1038/s43018-021-00261-1

[52] Oren Y , Tsabar M , Cuoco MS , Amir‐Zilberstein L , Cabanos HF , Hütter JC , et al. Cycling cancer persister cells arise from lineages with distinct programs. Nature. 2021;596(7873):576–582. 10.1038/s41586-021-03796-6 34381210 PMC9209846

[53] Stein‐O'Brien G , Kagohara LT , Li S , Thakar M , Ranaweera R , Ozawa H , et al. Integrated time course omics analysis distinguishes immediate therapeutic response from acquired resistance. Genome Med. 2018;10:1–22. 29792227 10.1186/s13073-018-0545-2 PMC5966898

[54] Brady SW , McQuerry JA , Qiao Y , Piccolo SR , Shrestha G , Jenkins DF , et al. Combating subclonal evolution of resistant cancer phenotypes. Nat Commun. 2017;8(1):1231. 10.1038/s41467-017-01174-3 29093439 PMC5666005

[55] Jiménez‐Salazar JE , Damian‐Ferrara R , Arteaga M , Batina N , Damián‐Matsumura P . Non‐genomic actions of estrogens on the DNA repair pathways are associated with chemotherapy resistance in breast cancer. Front Oncol. 2021;11:1–10. 10.3389/fonc.2021.631007 PMC8044931 33869016

[56] Cho E , Montgomery RB , Mostaghel EA . Minireview: SLCO and ABC transporters: a role for steroid transport in prostate cancer progression. Endocrinology. 2014;155:4124–4132. 25147980 10.1210/en.2014-1337 PMC4298565

[57] Gavert N , Zwang Y , Weiser R , Greenberg O , Halperin S , Jacobi O , et al. Ex vivo organotypic cultures for synergistic therapy prioritization identify patient‐specific responses to combined MEK and Src inhibition in colorectal cancer. Nat Cancer. 2022;3:219–231. 35145327 10.1038/s43018-021-00325-2

