# Supplementary material for: Chemoresistome mapping in individual breast cancer patients unravels diversity in dynamic transcriptional adaptation
Source: Mol Oncol. 2025 Apr 28;19(12):3665–84. doi: 10.1002/1878-0261.70030 (PMC12688168; doi:10.1002/1878-0261.70030)
Supplement: Supplementary file 1 — Fig. S1. Flow chart of patients and samples used in the analysis. Fig. S2. Correlation between expression levels and scores of pathological markers. Fig. S3. Similarity between normal breast tissue from healthy individuals and adjacent normal tissue from cancer patients. Fig. S4. Temporal modulations in deregulation scores per‐patient for representative pathways. Fig. S5. Diverged temporal expression patterns associated with resistance Fig. S6. . Genes with shared pattern dynamics across patients Fig. S7. Pattern classification by correlating genes to theorethical patterns. Fig. S8. Kaplan–Meier curve of recurrence free survival for the entire cohort by their MP response score. Fig. S9. Distinct clustering of resistance genes in adjacent normal and posttreatment tumor tissues. Fig. S10. Hubs of resistance genes in selected dysregulated pathways. Fig. S11. Heat maps presenting the modes of resistance/reregulation in two representative dysregulated pathways. Fig. S12. Resolving resistance by pattern analysis of matched three timepoints. [file MOL2-19-3665-s007.pdf]

Chemoresistome Mapping in Individual Breast Cancer Patients Unravels Diversity in Dynamic Transcriptional Adaptation

Dadiani M et al.

Supplementary Figures

- **Figure S1:Flow chart of patients and samples used in the analysis**
- **Figure S2: Correlation between expression levels and scores of pathological markers**
- **Figure S3: Similarity between normal breast tissue from healthy individuals and adjacent normal tissue from cancer patients**
- **Figure S4: Temporal modulations in deregulation scores per-patient for representative pathways**
- **Figure S5:Diverged temporal expression patterns associated with resistance**
- **Figure S6: Genes with shared pattern dynamics across patients**
- **Figure S7: Pattern classification by correlating genes to Theoretical Patterns**
- **Figure S8: Kaplan-Meier curve of recurrence free survival by MP response score**
- **Figure S9: Distinct clustering of resistance genes in adjacent normal and post-treatment tumor tissues.**
- **Figure S10: Hubs of resistant genes in selected dysregulated pathways**
- **Figure S11: Heat maps presenting the modes of resistance/reregulation in two representative dysregulated pathways**
- **Figure S12: Resolving resistance by pattern analysis of matched three time points.**

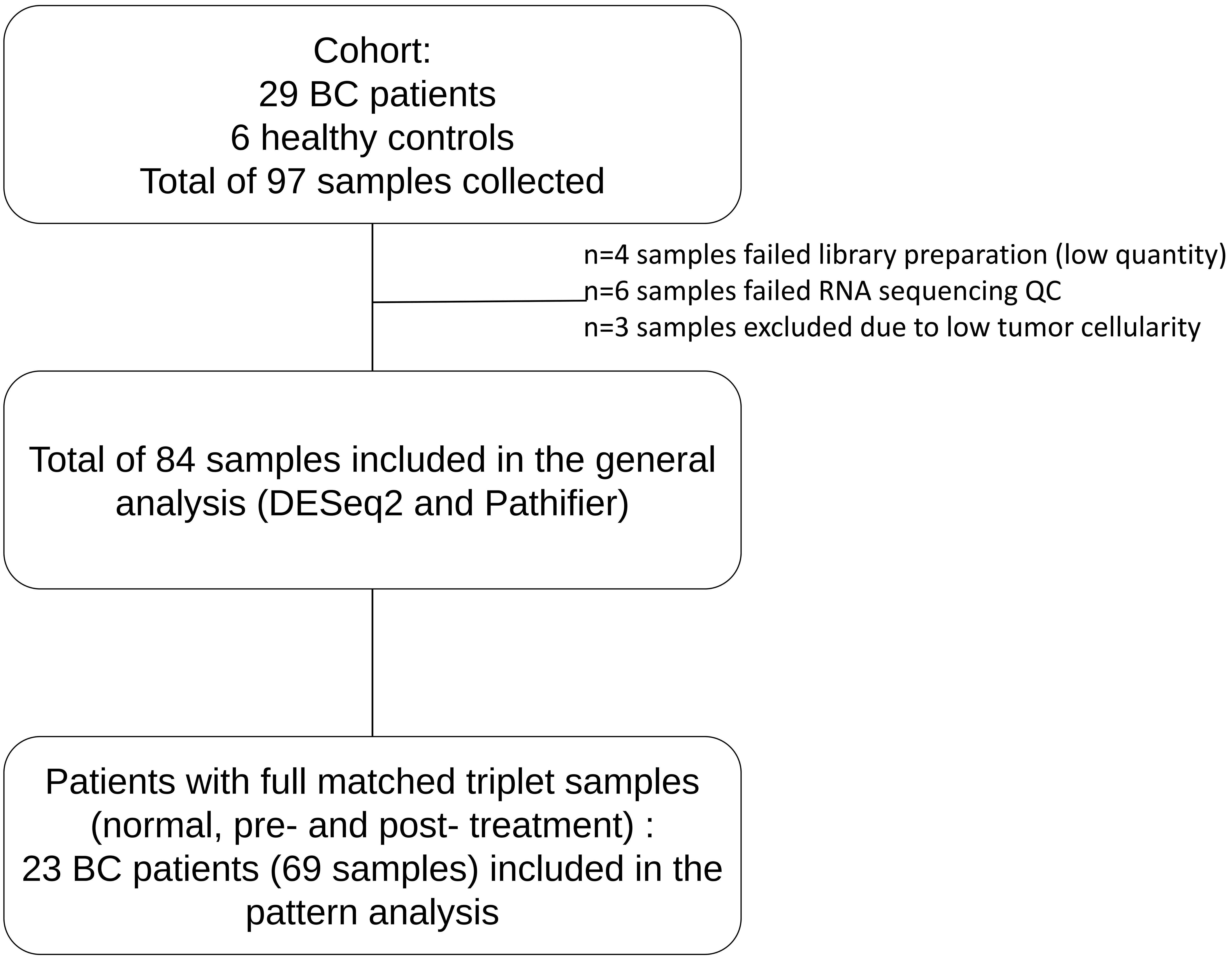

Figure S1: Flow chart of patients and samples used in the analysis

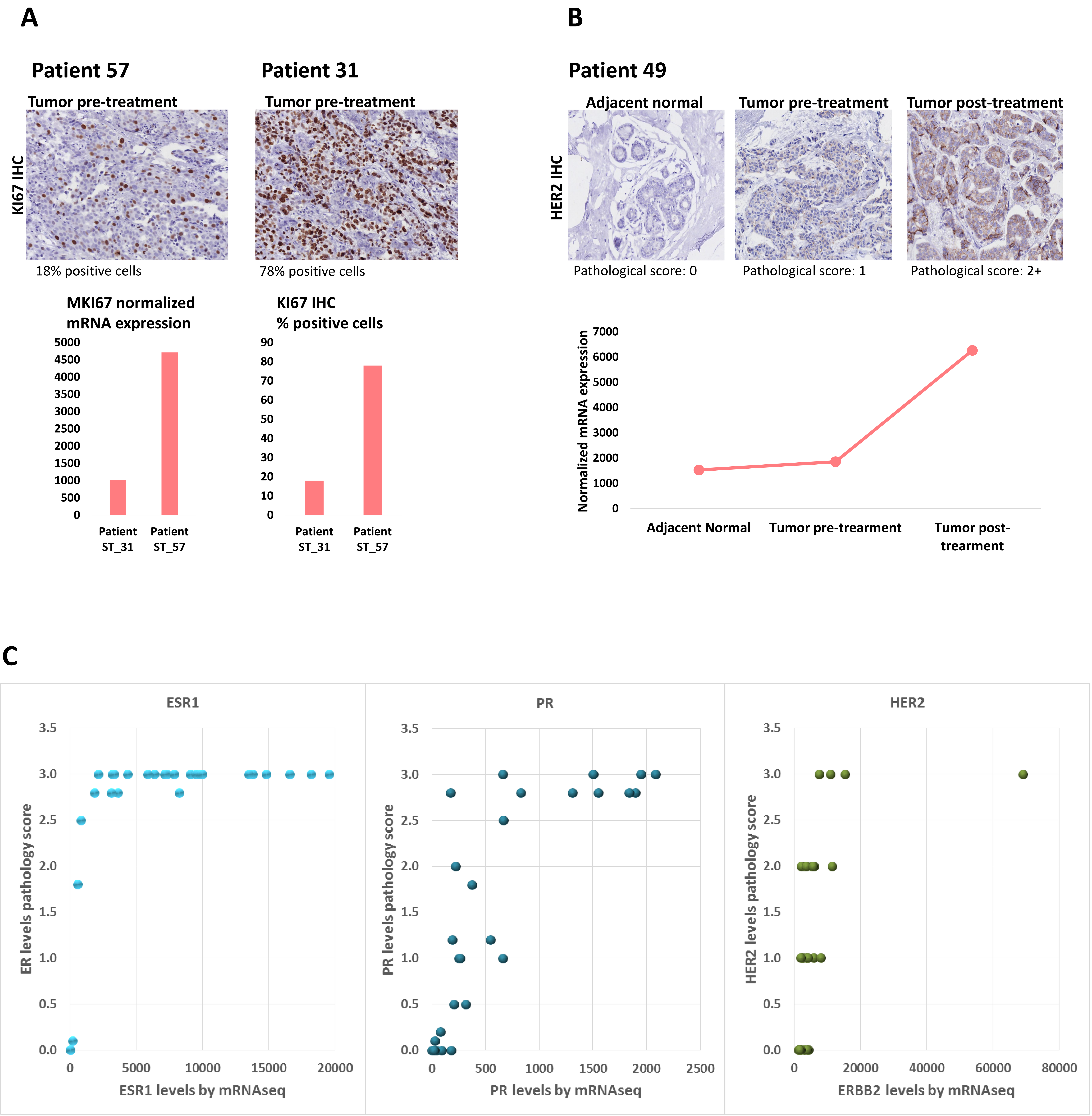

Figure S2. Correlation between expression levels and scores of pathological markers. A. MKI67 expression (normalized counts) of patients 57 and 31 are shown in red and green lines, respectively, for the three samples (adjacent normal, pre-treatment and post-treatment; x-axis). Percentage of Ki67 positive cells were assessed blinded to the expression data using the automated Virtuoso image analysis algorithm. B. ERBB2 expression levels (normalized counts and matched HER2 immunohistochemistry staining and its corresponding pathological score). C. The pathology score for each pathological marker is plotted against the normalized counts (RNA Seq) for the proteins ESR1 (left), PR (middle) and HER2 (right).

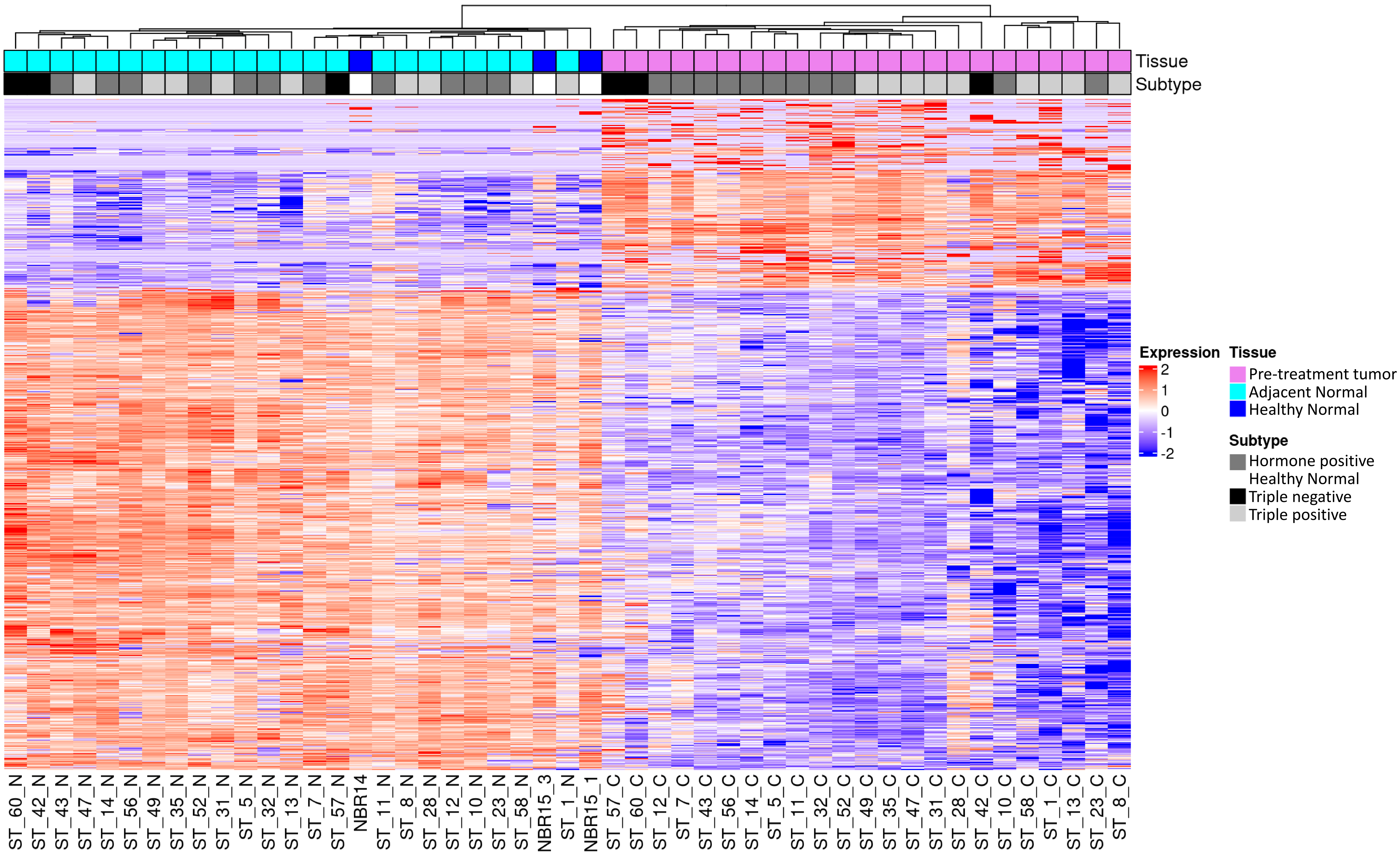

Figure S3: Similarity between normal breast tissue from healthy individuals and adjacent normal tissue from cancer patients. Heatmap showing a pairwise comparison of tumor samples (pre-treatment) versus normal breast tissue samples. The analysis includes the top 1,000 genes, differentiating tumor and normal samples, with an absolute log2 fold change greater than 1, ranked by adjusted p-value. Expression levels are presented as standardized log2 DESeq2 normalized counts. Adjacent normal breast tissue from cancer patients clusters closely with normal breast tissue from healthy individuals undergoing breast reduction, highlighting their similarity.

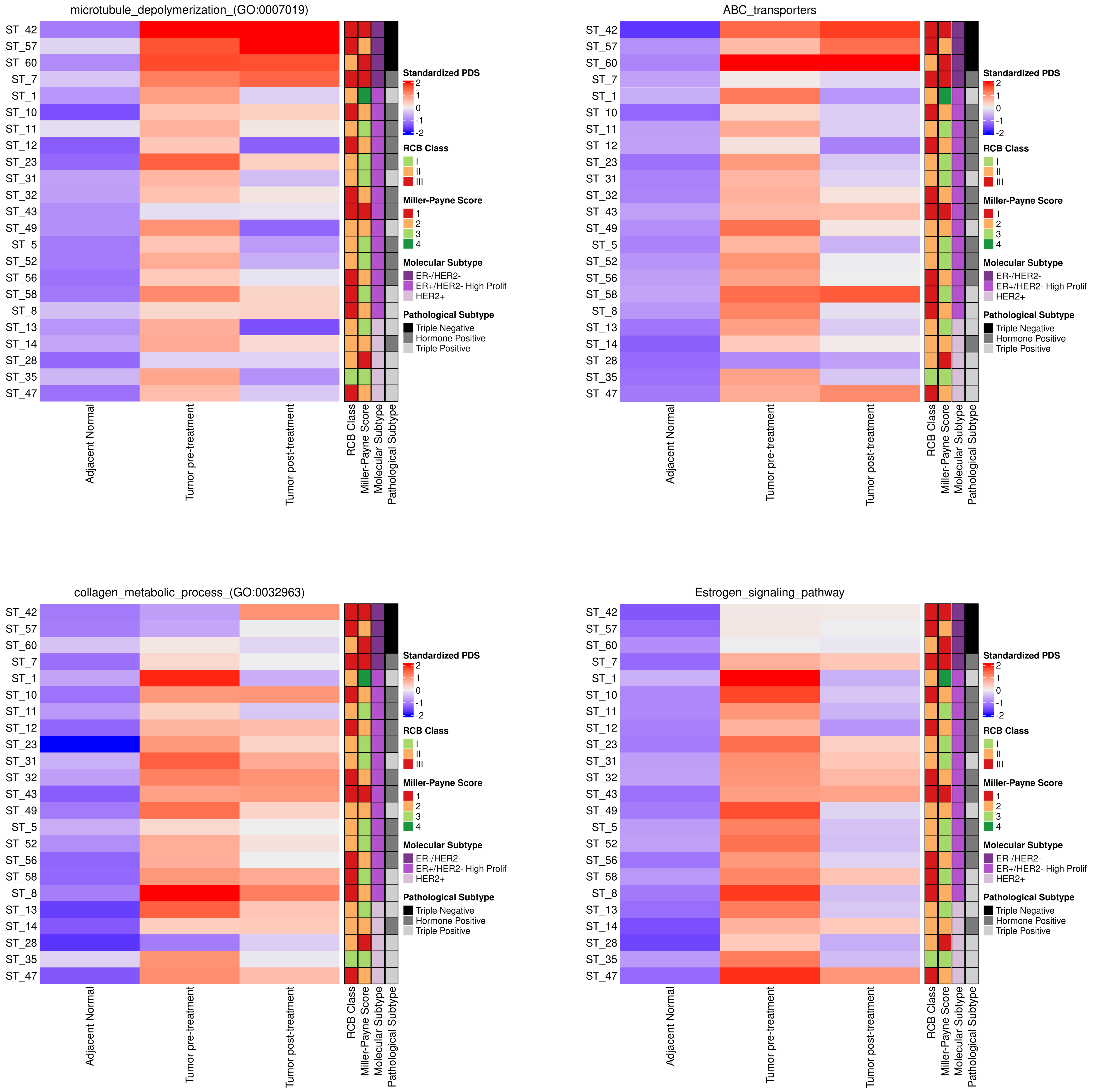

Figure S4: Temporal modulations in deregulation scores per-patient for representative pathways. Longitudinal representation of the pathway deregulation scores (PDS) calculated by Pathifier [43]. A heatmap of the PDS values, standardized to have for each patient zero mean and unit standard deviation. Each row relates to a patient. The columns relate to the tissue type.

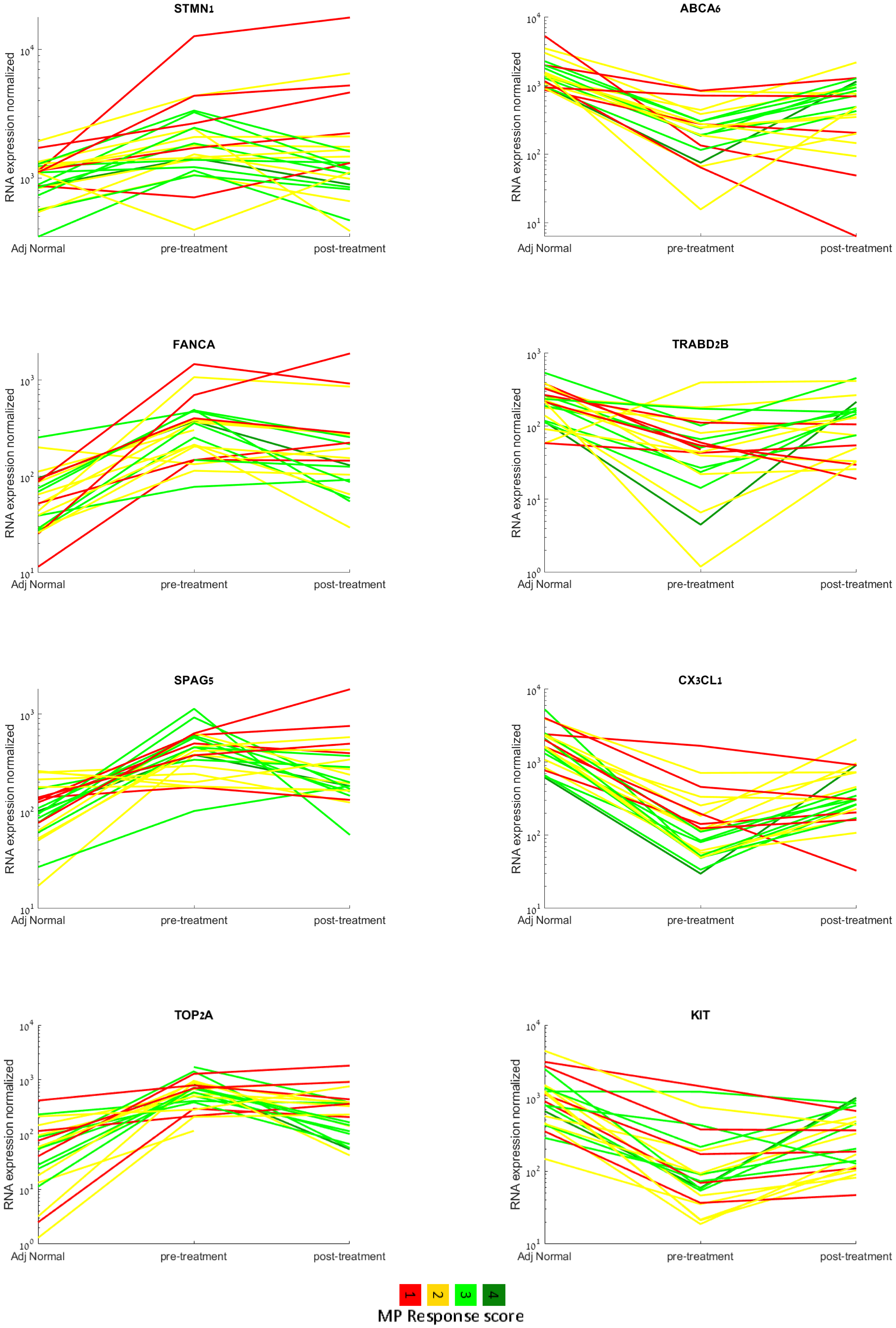

Figure S5: Divergent temporal expression patterns associated with resistance. Bimodal expression dynamics of representative genes that were significantly associated with pathological MP response score (by Wilcoxon Rank sum test).

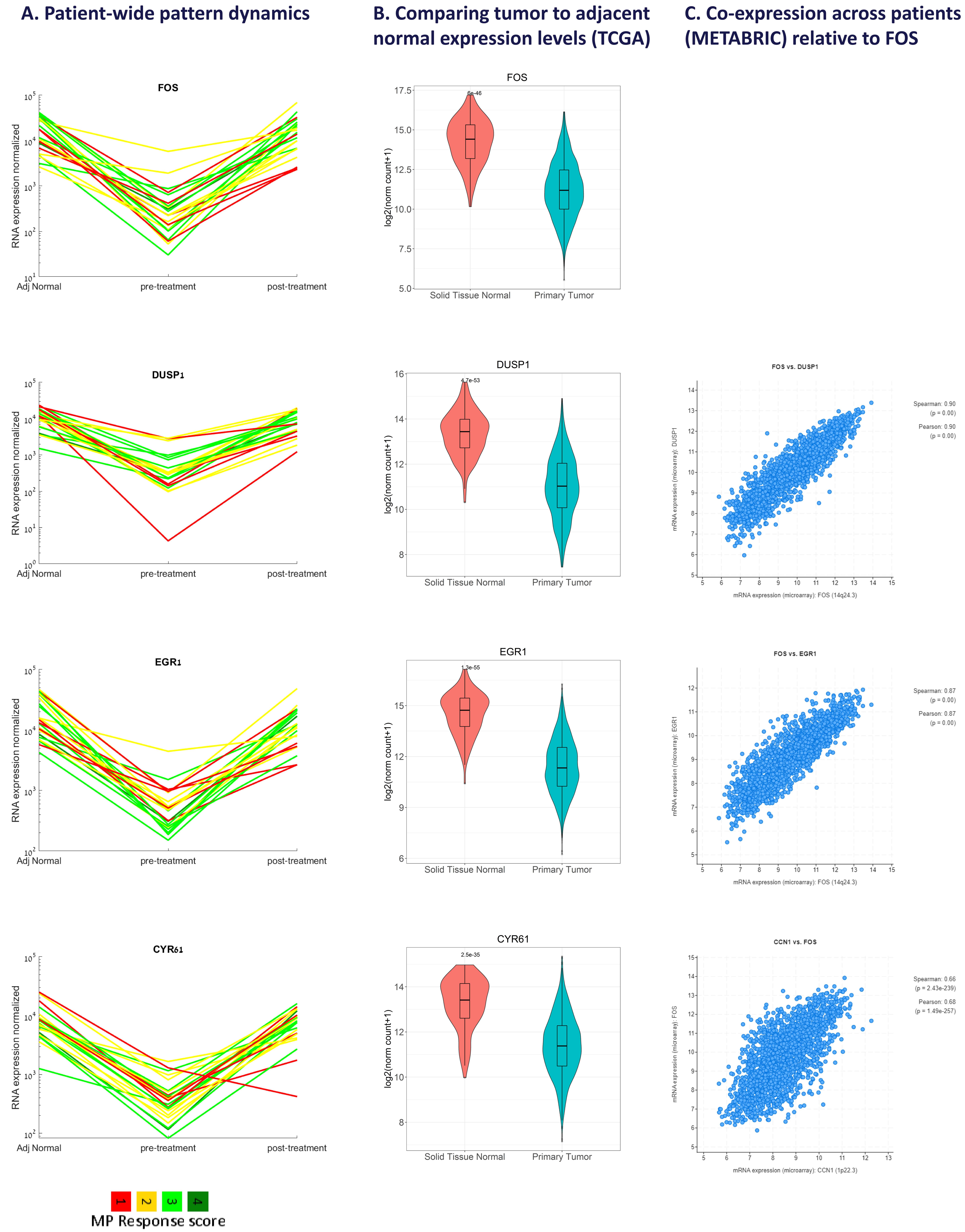

Figure S6: Genes with shared pattern dynamics across patients. A. Few genes exhibited the same pattern across all patients, independent of response or subtype. Each line denotes one patient, colored by the MP response score. B. Comparing expression values (log2(norm_counts+1) of tumor samples (n=1101) and adjacent normal samples (n=139). TCGA of breast cancer data downloaded from the XENA server. Adjusted p values (FDR corrected) are presented for each gene. C. These genes were found to have the most significant co-expression correlations across patients in large datasets (both TCGA and METABRIC). co-expression plots are derived from METABRIC dataset and plotted via cbioportal.

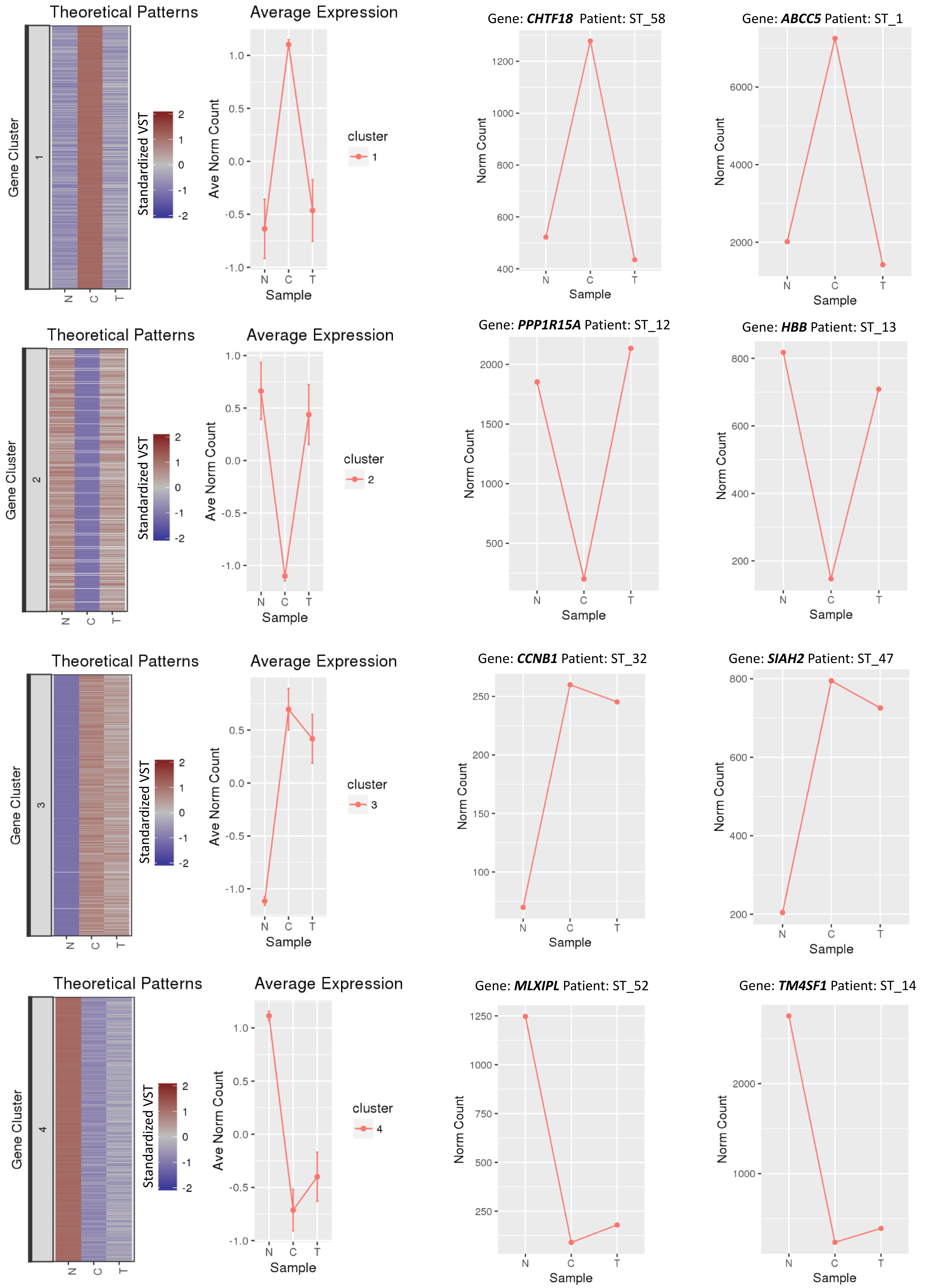

Figure S7. Pattern classification by correlating genes to Theoretical Patterns . The variance stabilized transformed (VST) expression values were correlated to the eight theoretical patterns**.** Each gene was assigned to a theoretical pattern (according to the maximal correlation). The left heatmap presents the expression for all the gene-patient profiles that were assigned to the pattern in the three tissue types (N, C and T). Shown are VST expression values, standardized to have for each gene zero mean and unit standard deviation. The expression profile is accompanied by a colored bar indicating the standardized values. The line plot shows the average expression of the standardized VST values. The error bars represent the standard deviation. On the right, shown are line plots of the Normalized counts of a gene in a single patient, in the three sample types (N, T and C) for two representative genes that were assigned to each of the patterns (P1-P4).

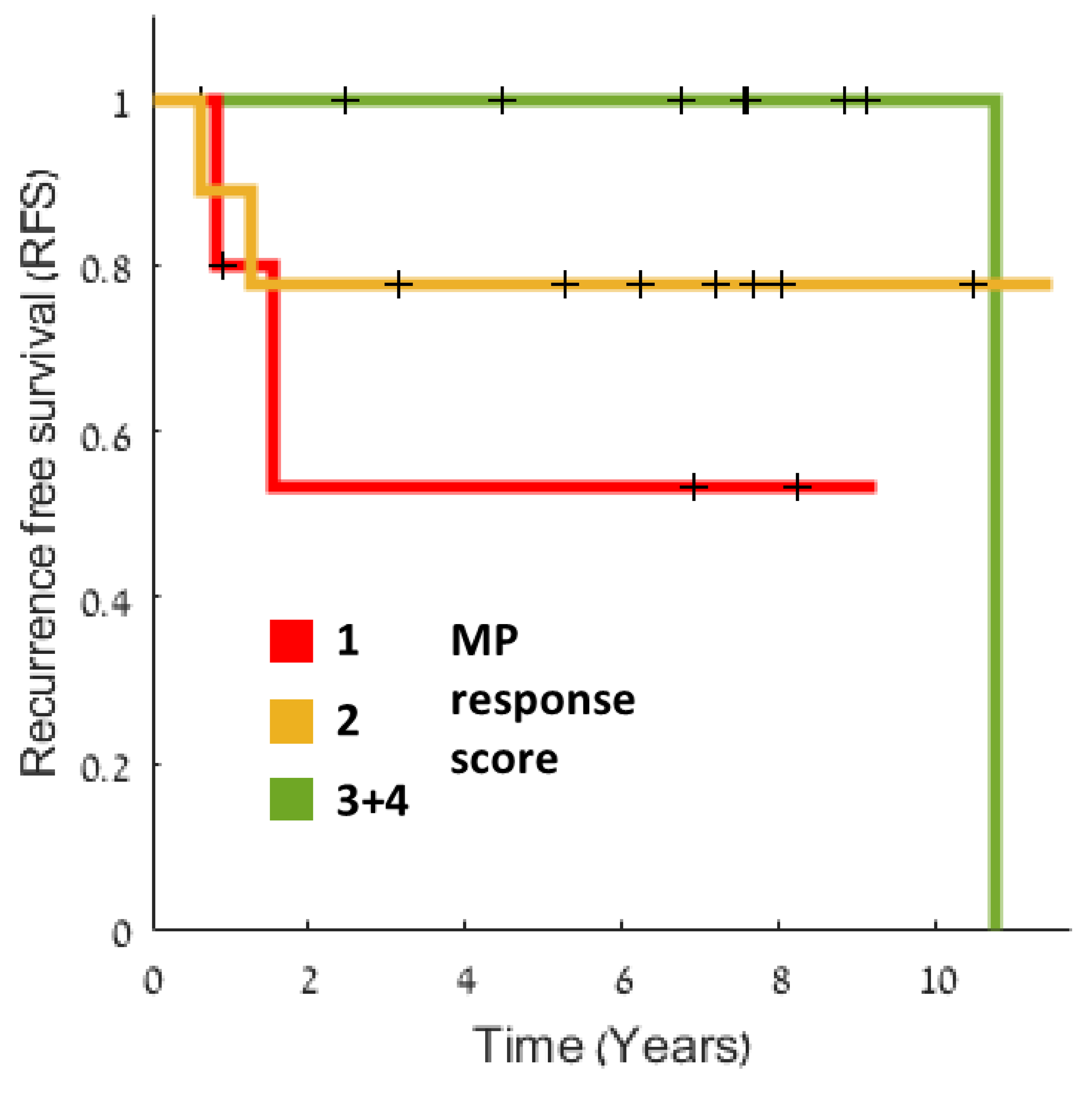

Figure S8: Kaplan-Meier curve of recurrence free survival for the entire cohort by their MP response score (Log rank p-value 0.16).

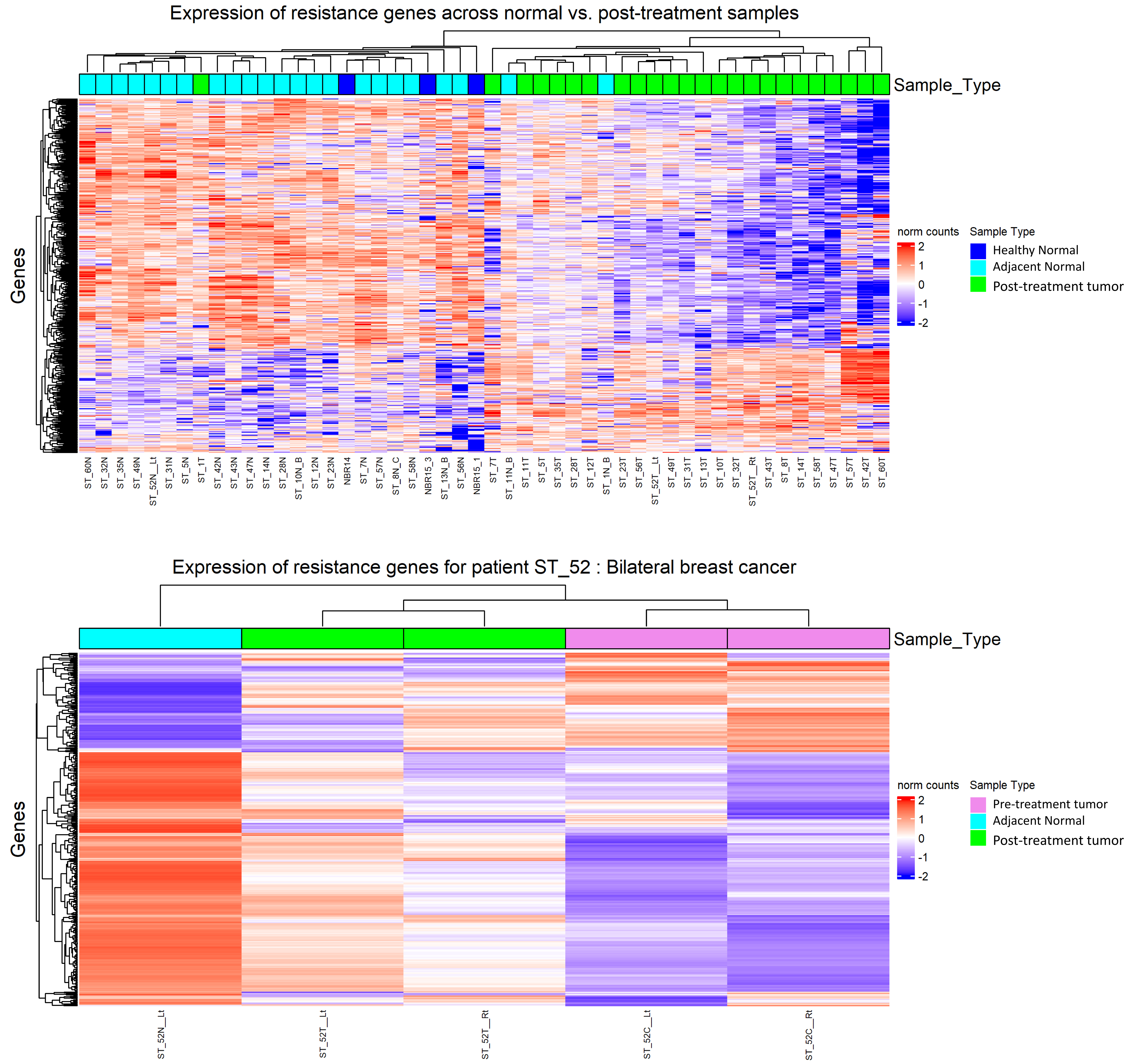

Figure S9: Distinct clustering of resistance genes in adjacent normal and post-treatment tumor tissues.

A. Heatmap depicting expression patterns of 452 resistance-associated genes primarily altered by treatment. Expression levels are represented as standardized log2 DESeq2 normalized counts. Pairwise comparison between post-treatment tumor samples and normal breast tissue samples is shown. Adjacent normal breast tissue from cancer patients clusters with healthy normal breast tissue, distinct from post-treatment tumors, indicating minimal treatment effects on adjacent normal tissues.

B. Focused view of the heatmap in (A), highlighting five samples from a single patient with bilateral breast cancer. This subset further illustrates the clear separation between adjacent normal tissue and post-treatment tumor samples.

Color scale represents standardized expression levels, with red indicating higher expression and blue indicating lower expression.

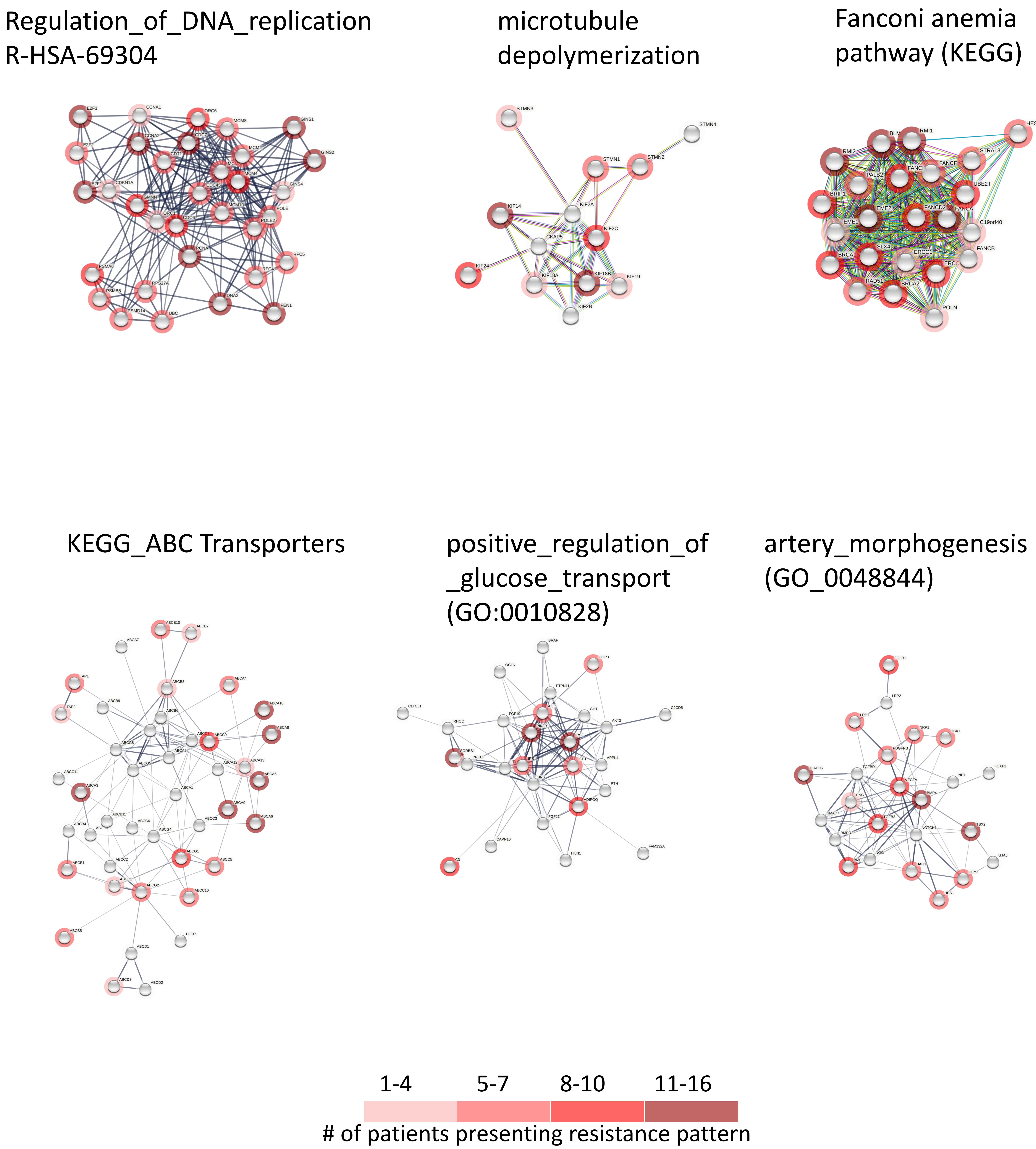

Figure S10: Hubs of resistant genes in selected dysregulated pathways. Network representation (STRING) of selected pathways. Genes are colored by the number of genes with resistant patterns.

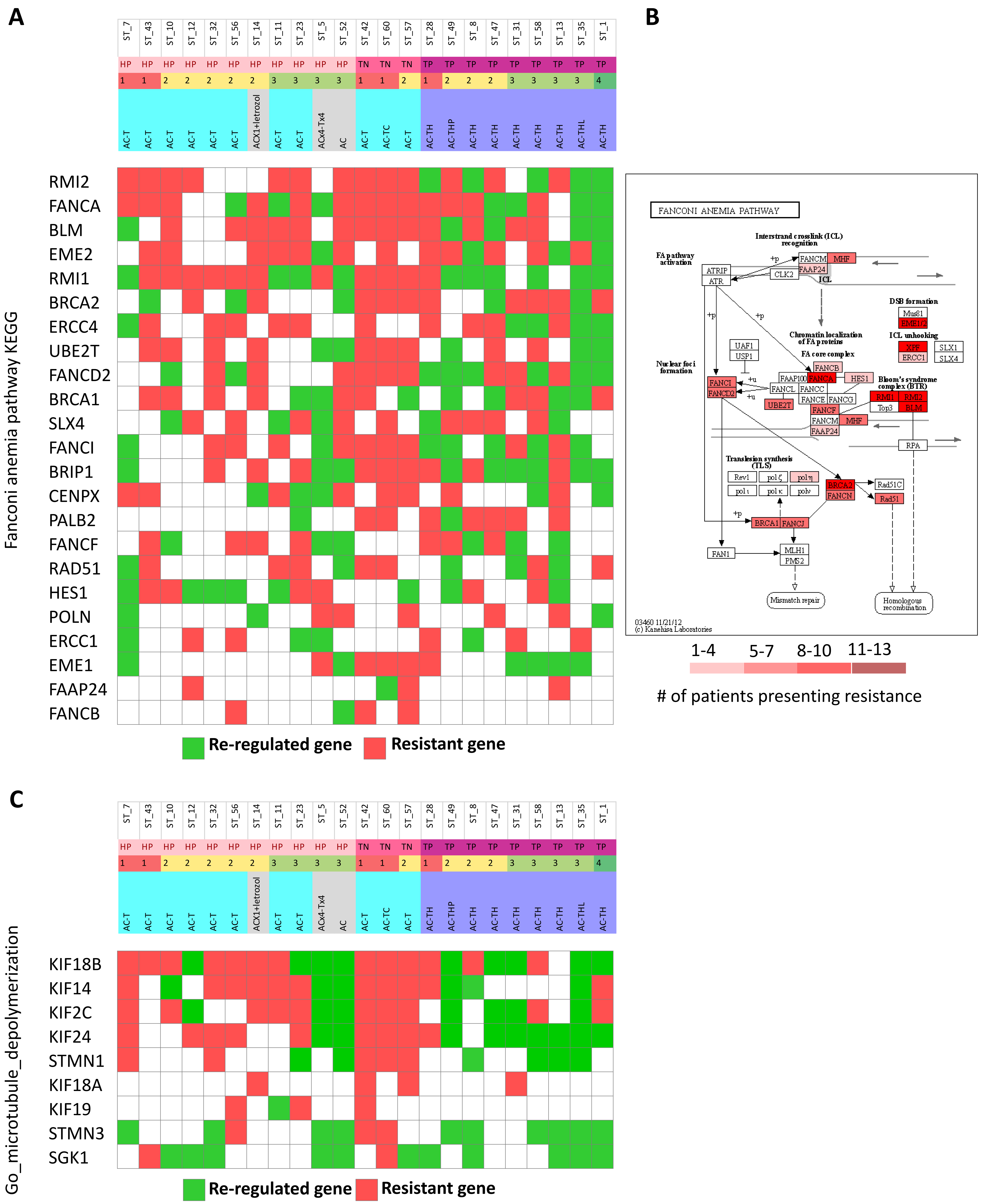

Figure S11: Heat maps presenting the modes of resistance/reregulation in two representative dysregulated pathways. A. All differentially expressed genes in the Fanconi Anemia pathway (KEGG) are colored by either the gene pattern was re-regulated (green) or resistant (red). Clinical parameters are presented for each patient, including subtype, response score and administered chemotherapy. B. A scheme of the Fanconi Anemia pathway (KEGG) colored by the number of patients presenting resistance pattern in each gene C. All differentially expressed genes in the Go_microtubule_depolymerization pathway and their mode of resistance or re-regulation in each patient. . TN – Triple-negative; HP – Hormone positive; TP – triple positive;

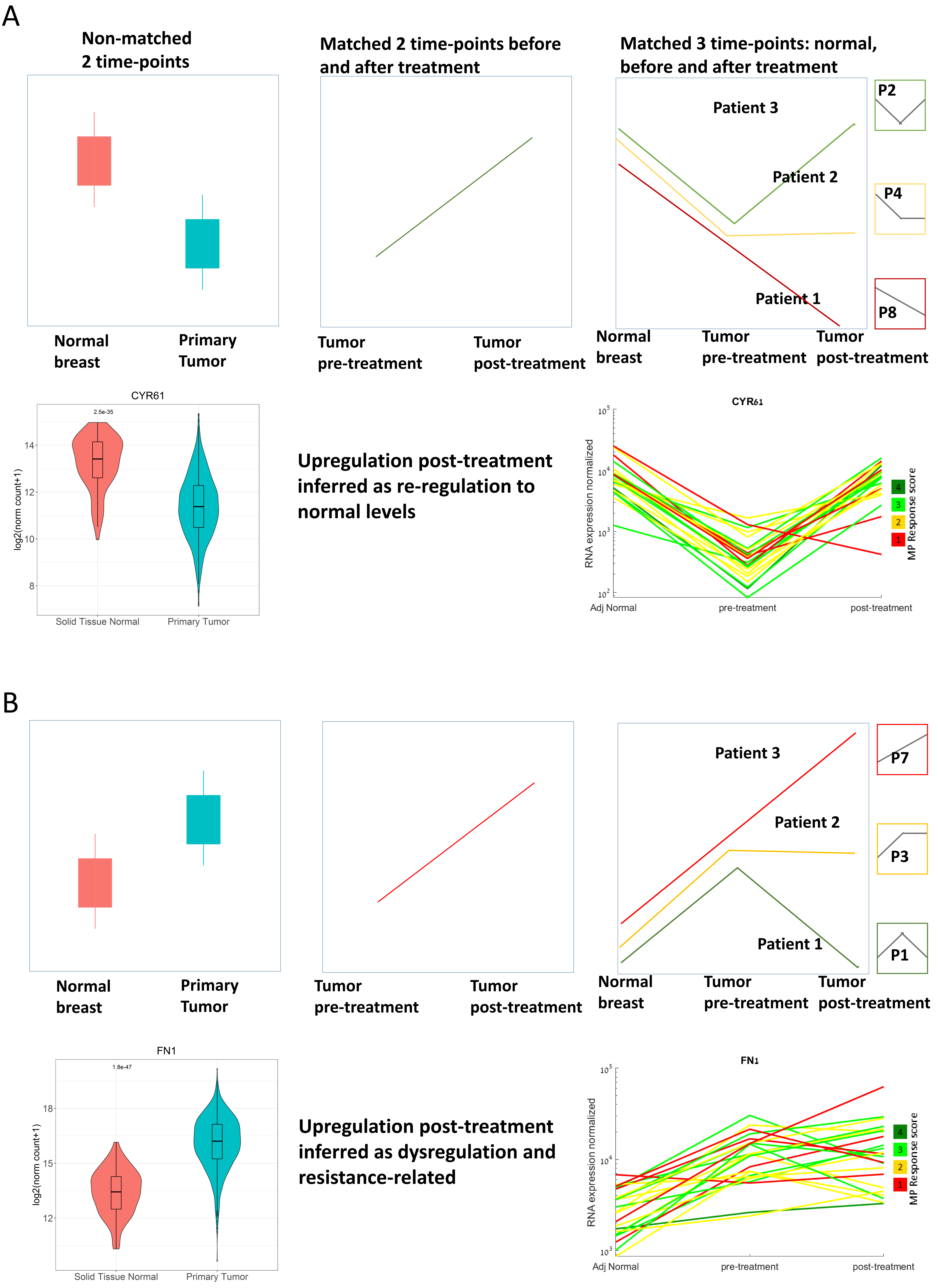

Figure S12: Resolving resistance by pattern analysis of matched three time points. Two theoretical scenarios and data examples of genes that are upregulated post treatment, but their normal levels relative to the tumor are opposite. pattern analysis enables to differentiate between interpretation of resistance vs. re-regulation.
